# Estimating effects of whole grain consumption on type 2 diabetes, colorectal cancer and cardiovascular disease: a burden of proof study

**DOI:** 10.1186/s12937-024-00957-x

**Published:** 2024-05-14

**Authors:** Houpu Liu, Jiahao Zhu, Rui Gao, Lilu Ding, Ye Yang, Wenxia Zhao, Xiaonan Cui, Wenli Lu, Jing Wang, Yingjun Li

**Affiliations:** 1 Department of Epidemiology and Health Statistics, School of Public Health, Hangzhou Medical College, 481 Binwen Road, Hangzhou, 310053 China; 2 Department of Radiology, Key Laboratory of Cancer Prevention and Therapy, Tianjin Medical University Cancer Institute & Hospital, National Clinical Research Center for Cancer, Tianjin, China; 3 Department of Epidemiology and Health Statistics, School of Public health, Tianjin Medical University, Tianjin, China

**Keywords:** Whole grains, Bayes Theorem, Cardiovascular diseases, Diabetes Mellitus, type 2, Colorectal neoplasms

## Abstract

**Background:**

Previous studies on whole grain consumption had inconsistent findings and lacked quantitative assessments of evidence quality. Therefore, we aimed to summarize updated findings using the Burden of Proof analysis (BPRF) to investigate the relationship of whole grain consumption on type 2 diabetes (T2D), colorectal cancer (CRC), stroke, and ischemic heart disease (IHD).

**Methods:**

We conducted a literature search in the Medline and Web of Science up to June 12, 2023, to identify related cohort studies and systematic reviews. The mean RR (relative risk) curve and uncertainty intervals (UIs), BPRF function, risk-outcome score (ROS), and the theoretical minimum risk exposure level (TMREL) were estimated to evaluate the level of four risk-outcome pairs.

**Results:**

In total, 27 prospective cohorts were included in our analysis. Consuming whole grain at the range of TMREL (118.5–148.1 g per day) was associated with lower risks: T2D (declined by 37.3%, 95% UI: 5.8 to 59.5), CRC (declined by 17.3%, 6.5 to 27.7), stroke (declined by 21.8%, 7.3 to 35.1), and IHD (declined by 36.9%, 7.1 to 58.0). For all outcomes except stroke, we observed a non-linear, monotonic decrease as whole grain consumption increased; For stroke, it followed a *J*-shaped curve (the greatest decline in the risk of stroke at consuming 100 g whole grain for a day). The relationships between whole grain consumption and four diseases are all two-star pairs (ROS: 0.087, 0.068, 0.062, 0.095 for T2D, CRC, stroke, and IHD, respectively).

**Conclusion:**

Consuming 100 g of whole grains per day offers broad protective benefits. However, exceeding this threshold may diminish the protective effects against stroke. Our findings endorse replacing refined grains with whole grains as the main source of daily carbohydrates.

**Registry and registry number for systematic reviews or meta-analyses:**

We have registered our research in PROSPERO, and the identifier of our meta-analyses is **CRD42023447345**.

**Supplementary Information:**

The online version contains supplementary material available at 10.1186/s12937-024-00957-x.

## Introduction

Whole grains have been widely endorsed as a superior substitute for primary energy and carbohydrate sources in daily dietary guidelines because of their high dietary fiber content and numerous bioactive compounds [1]. The Global Burden of Disease Study 2019 (GBD 2019) has reported that lower intake of whole grain accounted for 1,844,836 (95% uncertainty interval [UI]: 2,338,609–921,291) deaths and 42.5 million (53.2–17.5) disability-adjusted life years (DALYs) [2]. The large estimated burden demonstrated the importance of fully appreciating the relationship between whole grain consumption and potentially related health outcomes and of further improving the strength of evidence supporting the understanding of those relationships.

Increasing evidence has found that a high intake of whole grains is related to a reduction in the risk of type 2 diabetes (T2D), colorectal cancer (CRC), ischemic heart disease (IHD), and stroke [2, 3]. However, regarding CRC, T2D and IHD, previous studies, including dose-response meta-analysis or cohort studies, exhibit variations in their consumption ranges. This complicates the comparability and consolidation of evidence [3–6]. Besides, in relation to stroke, recent meta-analyses have presented inconsistent findings [7, 8]. Although there is an increasing body of evidence supporting the positive impact of consuming whole grains on health, the challenge lies in accurately estimating RR associated with varying levels of consumption. This limitation hinders the ability of decision-makers to fully comprehend the strength of the connection between consuming whole grains and various health outcomes.

Burden of proof risk function (BPRF) is a new meta-analysis method that can quantitatively estimate the level of risk closest to the null hypothesis [9]. Hitherto, most of meta-regression studies applied given fixed knots to fit the spline models or forced a log-linear assumption to simplify statistical analysis. However, such a method may limit their ability to capture the effects of whole grain consumption on health outcomes, as the relationship between increasing whole grain intake and its impact on health might not be straightforward: it could lead to slight decreases in positive effects, or it could even become harmful if the consumption of whole grains goes beyond a certain point [3, 10]. Unlike existing methods, BPRF relaxed the conventional assumption of a log-linear shape in risk functions, and instead applied a data-driven approach to determine the relationship of risk-outcome pairs using a quadratic spline. Thus, BPRF can help to identify the ‘true’ shape of the risk function [11]. In addition, existing methods, such as Grading of Recommendations, Assessment, Development and Evaluations (GRADE) or NutriGRADE, are commonly applied to assess the quality of the underlying evidence [12]. However, such methods are unable to extend to quantify variation in true effect size caused by bias from covariates or other limitations of the evidence [11]. Nevertheless, BPRF can synthesize available evidence in algorithm to calculate uncertainty inclusive of between-study heterogeneity.

To precisely quantify the health effects of whole grain consumption, a meta-regression analysis was conducted on the evidence from prospective cohort studies. This study focused specifically on four health outcomes (T2D, CRC, IHD, and stroke) linked to whole grain consumption, as reported by the GBD study [13].

## Methods

Our protocol has been registered in International Prospective Register of Systematic Reviews (PROSPERO, identifier: **CRD42023447345**). We followed a standard framework of Preferred Reporting Items for Systematic Reviews and Meta-Analyses (PRISMA) guideline to report our results [14].

### Search strategy and selection criteria

We searched data published in English in the MEDLINE and Web of Science for systematic review, and cohort studies from 1 January, 2000 to 12 June, 2023, using standard search strings (Supplementary Table 1). A reference list of included publications was also manually screened to identify additional cohort studies. Titles and abstracts were screened by two reviewers (H Liu and J Wang), with discrepancies being reconciled through consulting a third author (Y Li).

Only prospective observational studies (for both incidence and mortality) published in English were included. Studies should report a relative risk ratio (RR), odds ratio (OR) or hazard ratio (HR) of the associations between whole grain consumption and at least one of the four outcomes. Additionally, they should specify the amount of whole grain consumption in both the reference group and the alternate group for comparison.

Retrospective studies, conference abstracts, ecological studies, case reports, case-series, letters to the editor, conference proceedings, umbrella reviews, systematic reviews or meta-analyses as well as studies conducted in animals, children, or adolescents were excluded. Besides, we excluded studies that failed to report whole grain consumption without grams or servings equivalent, such as studies that used aggregated “diet scores” as a measure of consumption, and those that only reported specific subtypes of grains were also excluded. And studies reporting outcomes outside the scope of interest, such as all-cause mortality, or lacking specificity such as cardiovascular disease or diabetes mellitus, have been excluded.

### Data extraction

For each study, we collected the information of the eligible studies including the first author’s name, location, population characteristics (age, sex, race, and sample size), follow-up period, exposure definition, exposure assessment method, outcome definition, outcome ascertainment method, and covariates used in the study. Data were extracted by one author (H Liu) and checked by another author (J Wang) for accuracy. Besides, we also collected data on the range of exposure, sample size, person-years, number of events and risk estimate (RRs, HRs or ORs) and its corresponding uncertainty to conduct BPRF analysis. The uniform extraction procedures are shown in Supplementary Table 2.

We used a framework of BPRF methodology developed by Zheng et al. to assess the risk of bias in included studies [11, 15, 16]. For each included study, we extracted information concerning aspects of study design that could potentially bias the reported effect size and coded this information into study-level covariates [11]. These study-level covariates are followed as: follow-up time (≤ 10 months and > 10 months), exposure definitions, outcome definitions, effect size measures (HRs, RRs or ORs), the endpoint of outcome events (incidence or mortality), frequency of exposure measurements (single or repeat), outcome ascertainment methods (administrative records or self-reports), and the level of adjustment for relevant confounders (creating cascading dummy variables standing for the number of confounders adjusted in risk regression model from selected studies, and the minimum threshold for confounder adjustment for age and sex) [11]. These covariates would be further adjusted in our BPRF analysis if they significantly biased our estimated risk functions.

In addition to these covariates, we selected four common study characteristics that are highly relevant and likely to introduce bias, in order to evaluate the study quality [9, 17, 18]. These characteristics include the representativeness of the study population (whether it represents the general population or specific sub-groups such as high-risk populations), outcome confirmation, exposure mesurement and assessment, and control for confounding factors [11]. The quality score for each selected study was calculated by summing the scores across these four domains.

### Statistical methods

The estimates for our primary indicators of this work are mean RRs across a range of exposures, BRPFs, ROSs and star ratings for each risk-outcome pair. And the exposure unit was standardized to grams of consumption per day before synthesis. For each study that reported means or quantiles consumption rather than ranges of whole grain consumption, midpoint of defined quantile as the cutoff for intake intervals was used [10, 19–29]. When the quantile dose range didn’t have a specific endpoint, and mean and standard deviation weren’t available, we assumed a consumption level of 0 g per day as the lowest amount [22, 24, 25, 28, 30–33]. For the upper limit of consumption, we used the range from the closest quartile or tertile within the cohort. In addition, we used 30 g to evaluate one serving of whole grain consumption if the value of a serving was not stated [34].

### Estimating the shape of relationships between whole grain consumption and four health outcomes

We firstly modeled the mean log-RR (a measure of effect size) curve with MR-BRT (a Bayesian meta-regression tool) developed at the Institute for Health Metrics and Evaluation (IHME) [13, 35], and followed a uniform analysis procedure to select model specifications for all dietary risks, which is described by Zheng et al. [15]. For protective risk factors with hypothesis of monotonic deceresing, the final models were run applying quadratic splines with two internal knots and a linearity prior on the right tail [11, 15]. However, for the J-shaped risk curve, we employed quadratic splines with three internal knots and a linearity prior on both the right and left tails, without a monotonic prior [11]. Besides, to avoid the influence of extreme data and reduce publication bias, we trimmed 10% of data for each outcome as outliers [11].

Following the GRADE approach, we created binary covariates based on the the extracted information about specific study characteristics to identify potential sources of systematic bias within our included datasets. A step-wise Lasso approach were applied to assess the significance of these bias covariates at a threshold of 0.05. If the bias covariates were found to be significant, they were selected for adjustment in the final log-RR model.

To evaluate and adjust between-study heterogeneity, we quantified common sources of bias across the selected covariates that were likely to cause bias. And we calculated 95% UIs for each mean risk curve both with between-study heterogeneity incorporated (a ‘conservative’ UI) and without between-study heterogeneity incorporated (a ‘conventional’ UI) based on the selected biased covariates. Only the UIs that include between-study heterogeneity are presented in our main results unless specified.

Based on the aforementioned models, we then adjusted the selected bias covariates to decrease the variation in model residuals arising from differences in study quality and analysis. However, we primarily applied empirical evidence to choose bias covariate related to potential publication or reporting biases, which may ignore some confounders. Thus, Egger’s regression was conducted to detect publication bias, which estimated correlation between the study residuals and standard deviation of the corresponding data points. Funnel plots of the residuals of the risk function and standard deviations were generated to inspect reporting bias visually. And *P* value was used to assess the statistical significance of a risk for publication and/or reporting bias.

### Estimating the TMREL/minimum risk exposure level

To draw robust conclusions about health benefits of whole grain consumption, we calculated the theoretical minimum risk exposure level (TMREL) of all potential outcomes linked to consuming whole grains. TMREL aligns with real-world consumption patterns supported by the included data, enabling an estimation of the average risk associated with whole grain intake. For protective risk factors, the lower bound of TMREL is defined as the 85th percentile of the lower limit within the highest consumption range across all studies. whereas the upper bound of TMREL is determined as the 85th percentile of the midpoint within the highest consumption range across all studies [15].

### Estimating BPRF value, risk-outcome score (ROS) and star rating

Using the mean RR curves that incorporated between-study heterogeneity into uncertainty estimate, we estimated the BPRF from a conservative risk function. The BPRF was defined as the 5th (for harmful) or 95th percentile risk curve that is closest to the null. Afterwards, we calculated the ROS, which was equivalent to the mean log-BPRF averaged value over the 15th and 85th percentiles of the distribution of whole grain consumption. This value can give conservative interpretations regarding the association between whole grain consumption and four health outcomes [15, 36]. Then, the ROSs of risk-outcome pairs were converted into a comparison across risk-outcome pairs and a star rating (from one to five) was assigned based on the quantitative assessment of the association, where a one-star rating indicating a non-significant relationship based on the conservative interpretation, two-star through five-star ratings implying a decrease in risk with average exposure (compared to no exposure). And the ranges of ROS in 0–0.1398 stands for two-star pairs, > 0.1398–0.4055 for three-star pairs, > 0.4055–0.6152 for four-star pairs and greater than 0.6152 for five-star pairs for protective risks [11].

### Sensitivity analyses

To strengthen our estimates on the association between whole grain intake and four health outcomes and reduce the impact of outliers, we used trimming analysis with the Least Trimmed Squares (LTS) method. This method automatically identifies and removes outliers within the model’s likelihood. In our study, we trimmed the top 10% of data points that deviated the most from the expected dose-response curve as part of sensitivity analysis [11, 35].

Dose-response analysis on whole grain consumption and four health outcomes was conducted by applying MR-BRT tool which included several Python packages (limetr 0.0.5, mrtool 0.0.1, IPOPT 1.2.0). And we executed BPRF analysis in Visual Studio Code with extensions of R version 4.2.1 and Python 3.9.0.

## Results

### Study identification

A total of 3118 articles were found using search strings, and of those we identified 28 population-based prospective cohort studies, presenting a total of 184 estimates of effect sizes for associations between whole grain consumption and the four included health outcomes [10, 25, 26, 28, 29, 32, 33]. Details of the literature search are shown in Supplementary Fig. 1. Eleven studies were from the United States, including data mainly from the HPFS, NHS, NHSII, the ATBC cohort, and the Cancer Prevention Study‑II Nutrition Cohort [10, 20, 21, 27–29, 32, 38, 39, 41, 42]. Fifteen of 28 studies were from the European population [19, 22–26, 30, 33, 25, 43–47]. Besides, one study (the PURE cohort) collected information covering 21 countries (including the regions of North America and Europe, South America, Africa, the Middle East, South Asia, South East Asia, and China) [48] and one study reported the role of whole grain consumption on IHD in Chinese population [40]. Detailed information about the included cohorts is displayed in Supplementary Table 3.

### Characteristics of included studies

Of 28 included publications in the BPRF analysis, a total of eight studies investigated the association between whole grains and T2D [10, 28, 29, 32, 33, 37, 46, 47], seven for CRC [19–21, 30, 42–44], six for both IHD and stroke [23, 26, 39–41, 48], three for IHD [25, 38, 45], three for stroke [24, 27, 49], and one for IHD, stroke and CRC [22]. The median follow-up time of all included studies was 13.5 years (range: 6–25.8 years).

All included publications used dietary records or recalls, or food frequency questionnaires to collect data regarding whole grain intake. In total, seventeen publications used baseline data of whole grain intake in their analysis (single measurement) [19, 23–26, 30, 33, 39, 25, 43–45, 48], whereas ten considered the average whole grain intake throughout the follow-up (i.e., based on multiple measurements) as the main exposure [10, 20–22, 27, 37, 38, 40–42]. Three studies took self-report records to assess outcomes [28, 29, 32], and 25 studies used administrative medical records [10, 19, 21, 22, 25–27, 30, 33, 37, 41, 43, 44, 49]. Three studies used mortality as the endpoint [26, 39, 43], and the rest studies considered incidence as the endpoint. 8 studies reported effect sizes with RRs [19, 20, 24, 26, 30, 32, 33, 49], seventeen studies reported HRs [22, 23, 25, 27, 37–41, 48], one study reported ORs [46], and one study reported incidence rate ratios (IRRs) [44]. The detailed information is presented in Supplementary Tables 4–7.

### Estimation of the shape of whole grains with T2D, CRC, IHD and stroke

**Fig. 1:**
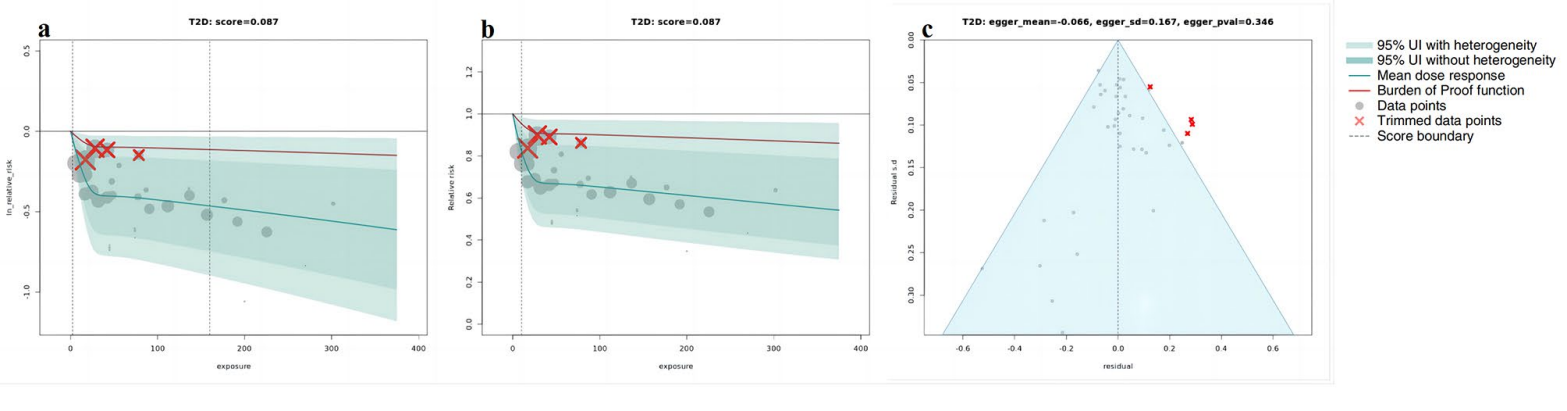
BPRF analysis on the association between whole grain consumption and T2D. a, log RR function. b, RR function. c, modified funnel plot showing the residuals (relative to zero) on the x-axis and the estimated s.d. that includes reported s.d. and between-study heterogeneity on the y-axis

**Fig. 2:**
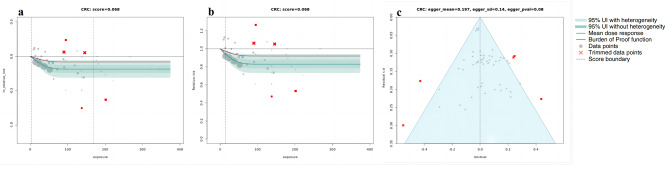
BPRF analysis on the association between whole grain consumption and CRC. a, log RR function. b, RR function. c, modified funnel plot showing the residuals (relative to zero) on the x-axis and the estimated s.d. that includes reported s.d. and between-study heterogeneity on the y-axis

**Fig. 3:**
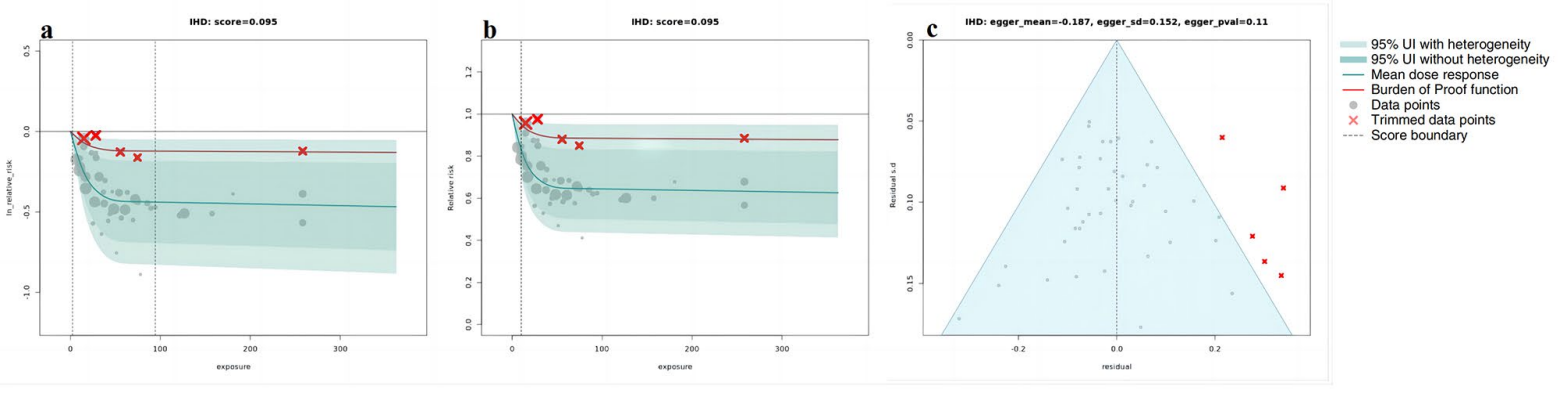
BPRF analysis on the association between whole grain consumption and IHD. a, log RR function. b, RR function. c, modified funnel plot showing the residuals (relative to zero) on the x-axis and the estimated s.d. that includes reported s.d. and between-study heterogeneity on the y-axis

**Fig. 4:**
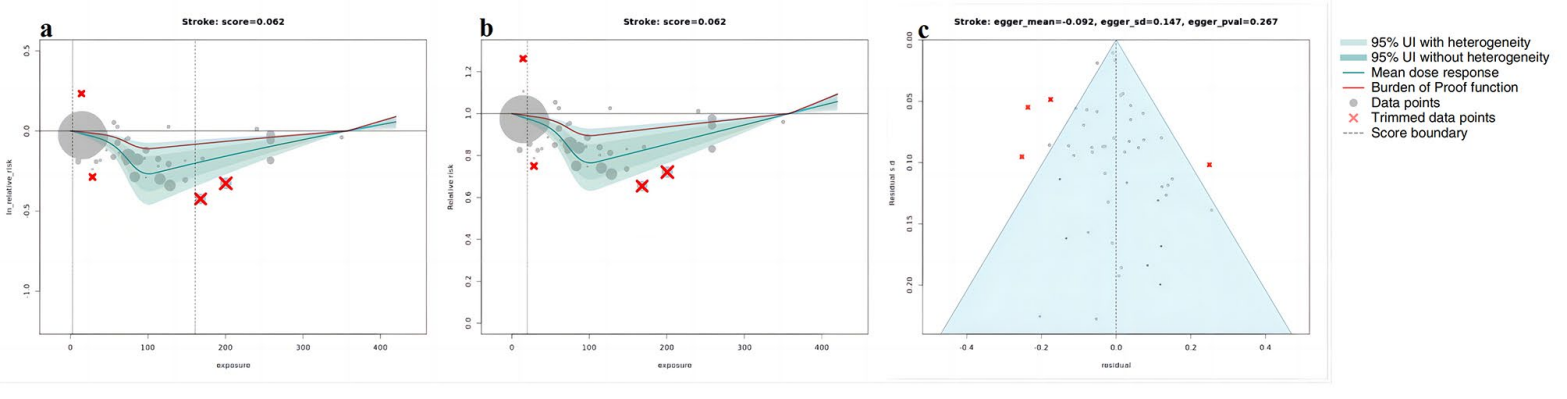
BPRF analysis on the association between whole grain consumption and stroke. a, log RR function. b, RR function. c, modified funnel plot showing the residuals (relative to zero) on the x-axis and the estimated s.d. that includes reported s.d. and between-study heterogeneity on the y-axis

Using BPRF methodology, our analyses revealed a correlation between higher whole grain intake and a reduced risk across all the outcomes considered. Figures 1, 2, 3 and 4 depict the BPRF curves for each risk-outcome pair, while Table 1 presents the results of the dose-response analysis.

**Table 1:** The relative risk at different levels of exposure, whole grains (g/day) and IHD, T2D, CRC, and stroke

Outcome	Intake level (g/day)	0	60	120	180	240	300	360
Type 2 diabetes	RR with conventional UI	1	0.67 (0.59, 0.74)	0.65 (0.57, 0.72)	0.62 (0.54, 0.70)	0.60 (0.51, 0.68)	0.58 (0.48, 0.65)	0.55 (0.46, 0.63)
	RR with conservative UI	1	0.65 (0.44, 0.95)	0.63 (0.41, 0.94)	0.62 (0.39, 0.94)	0.58 (0.35, 0.93)	0.56 (0.32, 0.93)	0.53 (0.29, 0.92)
Colorectal cancer	RR with conventional UI	1	0.84 (0.80, 0.88)	0.83 (0.79, 0.87)	0.83 (0.79, 0.87)	0.83 (0.79, 0.87)	0.83 (0.79, 0.87)	0.83 (0.79, 0.87)
	RR with conservative UI	1	0.84 (0.75, 0.95)	0.83 (0.73, 0.95)	0.83 (0.73, 0.95)	0.83 (0.73, 0.95)	0.83 (0.73, 0.95)	0.83 (0.73, 0.95)
Ischemic heart disease	RR with conventional UI	1	0.65 (0.58, 0.72)	0.64 (0.57, 0.71)	0.64 (0.57, 0.71)	0.64 (0.57, 0.71)	0.63 (0.56, 0.70)	0.63 (0.56, 0.70)
	RR with conservative UI	1	0.64 (0.43, 0.93)	0.63 (0.42, 0.93)	0.63 (0.42, 0.93)	0.62 (0.41, 0.93)	0.62 (0.40, 0.93)	0.61 (0.40, 0.93)
Stoke	RR with conventional UI	1	0.90 (0.87, 0.92)	0.78 (0.73, 0.83)	0.84 (0.80, 0.87)	0.89 (0.87, 0.92)	0.95 (0.93, 0.96)	1.00 (1.00, 1.00)
	RR with conservative UI	1	0.90 (0.83, 0.96)	0.77 (0.63, 0.92)	0.83 (0.72, 0.94)	0.89 (0.82, 0.96)	0.95 (0.91, 0.98)	1.01 (1.00, 1.01)

Specifically, our analysis revealed that the associations between whole grain consumption and the risk of T2D, CRC and IHD all exhibited non-linear, monotonically decreasing trends (Figs. 1, 2 and 3). In regard to T2D (Fig. 1a and b), the sharpest decline in risk was noted at daily consumption of 50 g, with a reduction of 34.3% (95% UI including between-study heterogeneity: 5.3 to 55.7), compared to no whole grain consumption (at 0 g per day). Nonetheless, the reduction in risk tapered off to a mere 1.2% (0.2–1.6) when comparing a consumption level of 90 g per day to 50 g per day. With respect to CRC (Fig. 2a and b), the largest reduction in CRC risk was identified when comparing the risk between an intake of 0 g per day and of 80 g per day, showcasing a noteworthy decline of 17.3%. we observed only marginal additional reductions in risk when consumption is beyond 80 g per day. As for IHD, the steepest decline of 32.1% (95% UI inclusive of between-study heterogeneity of 6.0 to 51.8) in IHD risk was observed when comparing risk between an intake of 0 g per day and of 30 g per day, with more modest marginal declines in IHD risk when consumption levels greater than 30 g per day (Fig. 3a and b).

Different from the aforementioned results, a *J*-shape association was found between whole grain consumption and the risk of stroke (Fig. 4a and b). The greatest reduction in stroke risk, observed at an intake of 100 g per day, was 24.6% (95% UI including between heterogeneity: 8.8 to 38.8). The mean risk of stroke at 60 g per day was 14.1% (7.0 to 21.3 including between-study heterogeneity) higher than at 100 g per day. And it was 1.5% (0.3 to 2.0) higher at 120 g per day compared to 100 g per day.

Additionally, the BPRF estimated ROSs for IHD, T2D, CRC and stroke of 0.095, 0.087, 0.068 and 0.062, respectively, which were applied to explore the average health benefits across the universe of whole grain consumption. Such estimates indicated that the consuming whole grains, on average, was related to a 9.9% decreased risk of IHD, a 9.1.% lower risk of T2D, a 7.0% lower risk of CRC and a 6.4% lower risk of stroke compared to a 0 g of whole grain intake. The star ratings of the four risk-outcome pairs all correspond to a two-star rating. After adjusting for between-study heterogeneity, the relationships still achieved statistical significance.

### TMREL level of whole grain consumption

Based on observed exposure levels reported in the included studies, a TMREL of 118.5 g to 148.1 g per day, corresponding to approximately 4–5 servings per day, was estimated (detailed input information presented in Supplementary Table 8). Compared to TMREL (118.5–148.1 g), consuming no whole grains was associated with a 37.3% (5.8 to 59.5, inclusive of between-study heterogeneity) greater mean risk of T2D, a 17.3% (95% UI inclusive of between-study heterogeneity of 6.5 to 27.7) greater risk of CRC, a 36.9% (95% UI inclusive of between-study heterogeneity of 7.1 to 58.0) greater mean risk of IHD, and a 21.8% greater mean risk of stroke (95% UI inclusive of between-study heterogeneity of 7.3 to 35.1).

### Sensitivity analysis and publication bias

The sensitivity analyses showed that trimming had significant effects on the ROS and reporting bias of the association between whole grain consumption and T2D and IHD. Without trimming, the ROS of T2D is -0.136, and a significant publication bias was detected using Egger’s regression (*P* = 0.023, as shown in Supplementary Fig. 2c). With respect to IHD, the results without trimming have reported a ROS of -0.273 and statistically significant evidence of small-study bias (*P* for Egger’s regression = 0.028, Supplementary Fig. 3c). However, both of the two health outcomes were found two significant study-level bias covariates (T2D: age of the population and outcome ascertainment methods; IHD: exposure measurement and outcome ascertainment methods). after removing outliers (T2D: 4 [31, 50], IHD: 5 [24, 50]) and adjusted for the selected bias covariates, no evidence of publication bias was observed (Figs. 1c and 3c). On the other hand, for CRC and stroke, trimming had a minor impact on the results. In both our analyses, with and without trimming, no significant evidence of publication bias was detected and no bias covariates were identified (Figs. 2c and 4c; and Supplementary Fig. 4c, 5c).

## Discussion

In this analysis, we applied a BPRF framework, which takes into account between-study heterogeneity, to quantify the association between whole grain consumption and four health outcomes. Our results suggested that increasing the intake of whole grains was significantly related to a reduction in the risk of CRC, T2D, IHD and stroke. When comparing TMREL (118.5–148.1 g per day) with a daily intake of 0 g of whole grains, the risk reductions for four diseases (T2D, CRC, IHD, and stroke) were 37.2%, 27.3%, 26.9%, and 21.8%, respectively. For all outcomes except stroke, we observed that mean risk exhibited a non-linear, monotonic decrease as whole grain consumption increased. However, the relationship between whole grains and stroke is like a *J*-shaped as the risk increased with exposure levels above or below a global minimum. Based on a conservative interpretation of available data (the averaged BPRF value), we found a slight decline in the risk of stroke, CRC, T2D, and IHD compared to no whole grain intake (by at least 6.4%, 7.0%, 9.1%, and 9.9%, respectively). The converted grade ratings of our evidence were all two-star ratings.

The protective role of whole grain consumption on CRC risk is well-documented. The World Cancer Research Fund/American Institute for Cancer Research (WCRF/AICR) has provided strong evidence on the protective role of whole grain consumption at 90 g/day on the risk of CRC (RR: 0.83; 95% CI: 0.79 to 0.89) [51]. Similarly, Schwingshackl et al. also reported an RR of 0.84 (95% CI: 0.78 to 0.90) of whole grain consumption at 90 g per day, and such evidence was rated as moderate according to the NutriGrade recommendation [5]. In consistent with previous studies, we found that consuming 90 g whole grains per day was also related with a similar reduction in the risk of CRC (RR: 0.83; 95% UI: 0.73 to 0.95), although the range of confidence interval was relatively wide (due to the consideration of between-study heterogeneity). Furthermore, as computing the mean across the universe of studies is appropriate to estimate the relationship between risk and outcome [52], we calculated the averaged BPRF value of 0.932, which is corresponding to a decline in the mean risk of CRC by 7.0%. Additionally, the star rating considering between-study heterogeneity of the whole grains-CRC pair is two-star. This estimate implied that the strength of the association evidence was relatively weak, in contrast to the WCRF/ARIC assessment, which categorized the evidence as “convincing” [11]. In fact, the extent of biases in nutritional epidemiological studies, including substantial residual confounding and selective reporting, can significantly impact the accuracy of health risks estimates related to the studied nutrients. Furthermore, the observational findings from prospective cohort studies exhibit considerable variation across different research endeavors [53]. Therefore, it is important to consider the strength of the association and employ a quantitative approach to assess consistency (that is, between-study heterogeneity) when evaluating evidence. Additionally, it is advisable to adopt a more conservative interpretation [54]. Our risk assessment indicated that increasing whole grain consumption can slightly reduce the risk of CRC, after correcting for biases due to factors such as study design, the representativeness of the study population, control for confounding, and so on.

With respect to IHD, the evidence stemming from previous meta-analyses has displayed a lack of consistency. For instance, Hu H et al. only found a linear association with 3 knots percentiles (25th, 50th, and 75th) selected [8]. However, Bechthold A, et al. provided evidence of a non-linear dose-response association (*P*_non-linearity_<0.001) for IHD using three fixed knots at 10%, 50%, and 90% through the total distribution of the reported intake [7]. The disparities in their findings could be potentially due to variations in the selection of different knot placements along the estimated risk function curve, which might influence on the resulting accuracy of a spline approximation of a curve [55]. On the other hand, BPRF analysis, according to the given degree and number of knots, automatically sampled a set of knot placements for a feasible knot distribution, evaluated each resulting model by computing its fit and curvature, and then aggregated the final model as a weighted combination of the ensemble to mitigate the effect of spline parameter selection results and draw a robust conclusion. With this methodology, we found a non-linear, monotonic decline association between whole grain consumption and IHD.

In the case of stroke, previous meta-analyses generated mixed results. Bechthold et al. observed no association between whole grain intake and the risk of stroke in the non-linear dose-response analysis [7]. Conversely, Aune et al. observed a protective role of whole grain consumption on stroke risk, but this role was only significant in their non-linear dose-response analysis, and the risk curve exhibited a *J*-shaped pattern [6]. The difference between these studies might be partially attributable to different included studies [6, 7]. Our analysis, including the results of newly published studies (the PURE study, China Kadoorie Biobank study and UK Biobank study) and applying BPRF methodology (free of log-linear hypothesis), found a *J*-shaped relationship between whole grain consumption and stroke, and we observed the greatest reduction in stroke risk observed at an intake of 100 g per day (RR: 0.75, 95%UI: 0.62 to 0.92). Unlike our analysis, both of the aforementioned studies assumed the association between whole grains and stroke to be log-linear [6, 7], which might be inappropriate. A log-linear association implies that a fixed increment of health roles of whole grain consumption (for example, 30 g/day) remains constant across all levels of intake; however, an increase in consumption from 0 to 120 g/day would not have the same impact as an increase from 240 to 360 g/day, especially considering that excessive consumption may cause health issues such as overweight [56].

Our analyses support the need for stronger efforts and policies to encourage increased whole grain consumption as a means to reduce the risk of chronic diseases. Whole grains are well-known for their abundance of dietary fiber and nutrients. However, they can also be a notable source of food-borne contaminants. Nonetheless, current evidence suggests that increasing whole grain consumption could improve public health [57]. We estimated a TMREL of 118.1–148.5 g per day as the high consumption levels of whole grain intake in the real world, and such estimates are in line with the recommended intake of whole grains promoted by the GBD and the World Health Organization (WHO) [58], which is at least 125 g per day [59]. To address both individual and environmental health, the Lancet EAT Commission recommends a primarily plant-based diet, including 232 g of whole grains per day to reduce the carbon footprint of animal-based foods [60]. Nevertheless, our analysis solely took the individual-level health benefits into consideration, and the potential environmental benefits of increased whole grain consumption were not evaluated. Based on our analyses, particularly the notable protective roles observed with daily consumption of 100 g of whole grains against the risk of stroke, it seems that incorporating a minimum of three servings of whole grains per day has the potential to lower the risk of chronic diseases.

Our study employed BPRF methodology to estimate the association between whole grain consumption and four health outcomes. Compared to traditional meta-analysis methods, this method could quantify between-study heterogeneity, and infer flexible risk functions. It does so without imposing a log-linear hypothesis, which may exaggerate risks at higher exposure levels and overlook crucial details at lower exposure levels. With this methodology, we have found that the risk curves for whole grain consumption and IHD, CRC and T2D displayed decreasing marginal returns, indicating that as whole grain intake increases, the incremental health benefits of whole grains decrease. In addition, quantifications of between-study heterogeneity and corrections for biases due to study design in the methods can contribute to a conservative interpretation and a better understanding of the protective role of whole grain consumption in real-world settings. Thirdly, by estimating RRs associated with consuming whole grains at the TMREL (in correspondence to high real-world consumption levels), we were able to provide sufficient evidence to justify more robust efforts and policies promoting increased whole grain consumption to reduce chronic disease risk, especially with regard to CRC, T2D, IHD and stroke. In general, our analysis results indicate that improving whole grain consumption is beneficial toward enhancing public health.

Although the methodological framework addressed by Zheng et al. overcame many of the limitations in existing meta-analysis approaches, this study still has several limitations. Firstly, all studies included in our analysis were observational, and we were unable to definitively assess causality. Besides, this study mainly focused on total or whole grain consumption, and the impacts of different specific subtypes of whole grains on health outcomes may vary. For example, previous reviews have indicated that increasing whole-grain breakfast cereals, other than whole-grain bread, may decrease the risk of stroke [6]; furthermore, oats or oatmeal are linked to lower all-cause mortality but show no impact on T2D and CVD incidence [6, 61]. Thus, further prospective cohort studies and randomized clinical trials focusing on different subtypes of whole grains and their associations with specific chronic diseases are required. Besides, most of the studies included were from the US and Europe, which limited the ability to make evidence-based recommendations, as dietary patterns can vary significantly between Asian and Western populations [62]. With respect to the Asian population, rather than whole grain consumption, most of studies investigated the role of refined grain consumption in the form of white rice and noodles [63], and further studies are needed to explore the association between whole grains and health outcomes on populations in Asia. Thirdly, the associations between whole grains and risks of different stroke types may be heterogeneous [24]. Unfortunately, we couldn’t investigate these associations separately due to a lack of reported data on stroke types in available studies.

## Conclusion

In conclusion, the present study demonstrates that the consumption of whole grains plays a protective role in the risks of CRC, T2D, IHD and stroke, and the BPRF analysis, which did not rely on log-linear assumptions, revealed non-linear associations between whole grain intake and the four diseases of interests. The star ratings converted by ROSs for all four outcomes are all two stars, indicating that the associations between whole grain intake and CRC, T2D, IHD and stroke remain significant. The current body of evidence justifies the need for increased efforts and policies to promote higher whole grain consumption for the betterment of public health.

### Electronic supplementary material

Below is the link to the electronic supplementary material.

Supplementary Material 1

## Data Availability

No datasets were generated or analysed during the current study.
