# Supplementary material for: Estimating effects of whole grain consumption on type 2 diabetes, colorectal cancer and cardiovascular disease: a burden of proof study
Source: Nutr J. 2024 May 14;23:49. doi: 10.1186/s12937-024-00957-x (PMC11092208; doi:10.1186/s12937-024-00957-x)
Supplement: Supplementary file 1 — Supplementary Material 1 [file 12937_2024_957_MOESM1_ESM.docx]

## Appendix - Estimating effects of whole grain consumption on type 2 diabetes, colorectal cancer and cardiovascular disease: a Burden of Proof study

**Section 1.**

We conducted literature searches to obtain input data from prospective studies evaluating the relationship between whole grain consumption and ischemic heart disease (IHD), stroke, type 2 diabetes (T2D), and colorectal cancer (CRC) in our analysis.

**Supplementary Table 1**. Search terms

| **Identification** | **Searching strings** |
| --- | --- |
| Whole grains | ("whole grains"[Mesh] OR "edible grain"[Mesh] OR "dietary fiber"[Mesh] OR "grain*"[tiab] OR "whole grain*"[tiab] OR "whole-grain"[tiab] OR "whole wheat bread"[tiab] OR "whole cereal"[tiab] OR "cereal*"[tiab] OR "cereal fiber*"[tiab] OR "cereal-fiber"[tiab] OR "breakfast cereal"[tiab] OR "dietary fiber"[tiab] OR "whole grain rice"[tiab] OR "whole-grain corn"[tiab] OR "food group*") |
| Colorectal cancer | ("colorectal neoplasm*"[Mesh] OR "colorectal neoplasm*"[tiab] OR "colon cancer*"[tiab] OR "colorectal cancer*"[tiab] OR "colorectal tumor*"[tiab] OR "stomach cancer"[tiab] OR "colorectal carcinoma"[tiab]) |
| Type 2 diabetes | ("Diabetes Mellitus, Type 2" [Mesh] OR "diabetes mellitus type 2" [tiab] OR "diabetes type 2" [tiab] OR "type 2 diabetes mellitus" [tiab] OR "type 2 diabetes" [tiab] OR "noninsulin dependent diabetes" [tiab] OR "adult-onset diabetes" OR "Diabetes Mellitus" OR "T2D"[tiab]) |
| Stroke | ("Ischemic Stroke" [Mesh] OR "ischemic stroke" [tiab] OR "ischaemic stroke" [tiab] OR "cerebral infarction" [tiab] OR "unspecified stroke" [tiab] OR "stroke" [tiab]) |
| Ischemic heart disease | ("Myocardial Ischemia" [Mesh] OR "Coronary Artery Disease" [Mesh] OR "Angina, Stable" [Mesh] OR "Acute Coronary Syndrome" [Mesh] OR "ischemic heart disease" [tiab] OR "ischaemic heart disease" [tiab] OR "coronary artery disease" [tiab] OR "coronary heart disease" [tiab] OR "myocardial ischemia" [tiab] OR "myocardial ischaemia" [tiab] OR "myocardial infarction" [tiab] OR "angina" [tiab] OR "acute coronary syndrome") |
| Prospective cohort | ("Cohort Studies"[Mesh] OR "Prospective cohort" [tiab] OR "case-cohort" [All Fields] OR "Follow-up" [All Fields] OR "Longitudinal" [tiab]) |
| Systematic review | ("Systematic Review" [Publication Type] OR "systematic review" [tiab] OR "Meta-Analysis" [Publication Type] OR "meta-analysis" OR "Dose-Response" [tiab]) |

**Supplementary Figure 1: Flow chart of literature search**


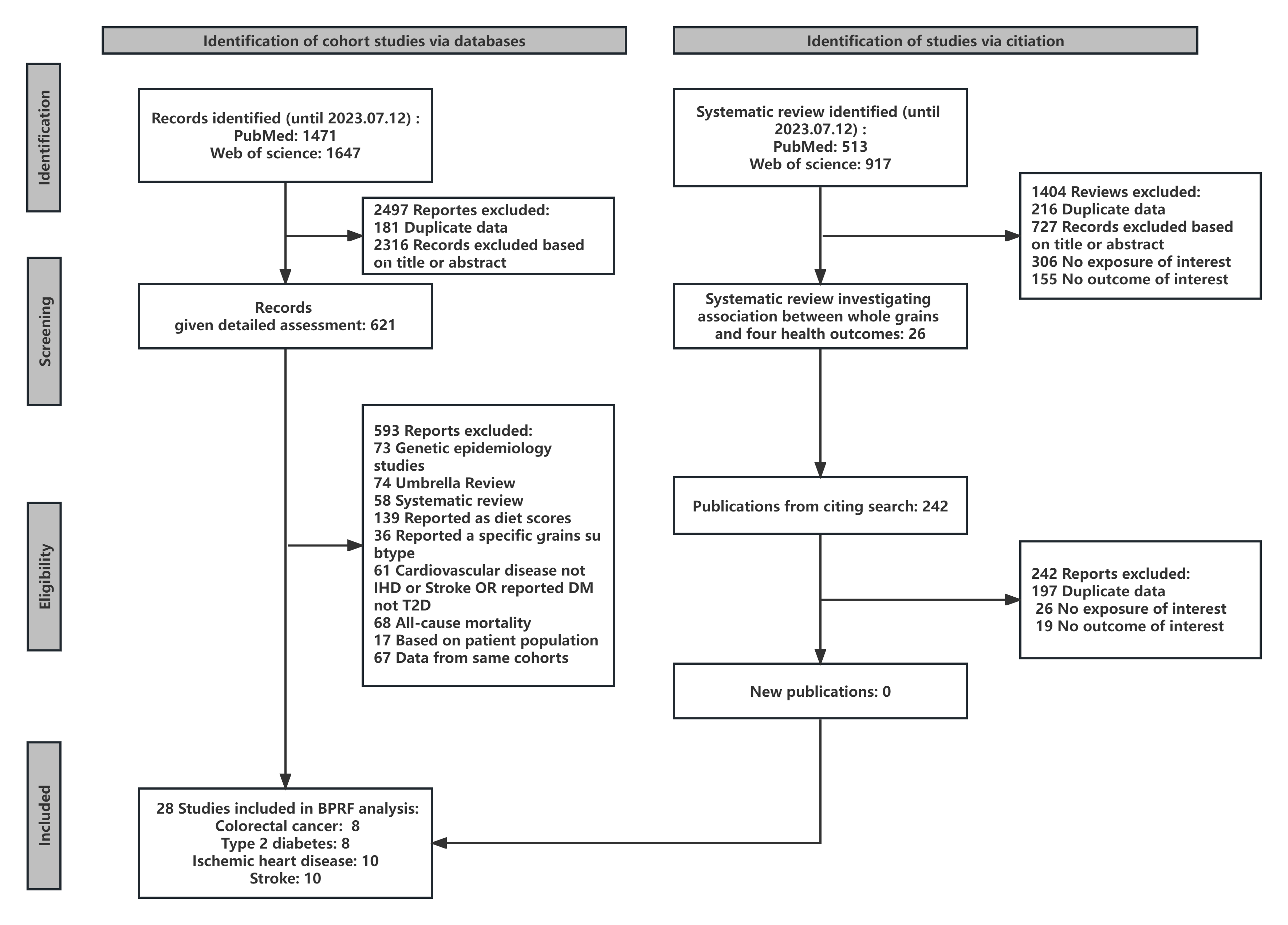


**Section 2.**

For each study that met the inclusion criteria, two reviewers assessed several indicators of bias during the extraction process. The full list of bias covariates assessed across all studies can be found in the extraction template

**Supplementary Table 2.** Causal criteria extraction template

| **Category** | **Type of value** | **nid** |
| --- | --- | --- |
| R-O pair | character | risk |
|  | character | risk_mapping |
|  | character | outcome |
|  | character | outcome_mapping |
| Location | character | location_name |
|  | integer | rep_geography |
|  | character | rep_selection_criteria |
|  | integer | rep_prevalent_disease |
| Study Population | integer | age_start |
|  | integer | age_end |
|  | integer | age_mean |
|  | integer | age_sd |
|  | integer | age_issue |
|  | double | percent_male |
|  | integer | sex_issue |
| Study Design | character | design |
|  | character | study_name |
| Exposure | character | exp_assess_level |
|  | character | exp_instrument |
|  | character | exp_assess_period |
|  | character | exp_assess_num |
|  | character | exp_method_1 |
|  | character | exp_method_2 |
|  | character | exp_method_3 |
|  | character | exp_recall_period |
|  | integer | exp_recall_period_value |
|  | character | exp_recall_period_other |
|  | character | exp_type |
| Outcome | character | outcome_def |
|  | character | outcome_type |
|  | character | outcome_assess_1 |
|  | character | outcome_assess_2 |
|  | character | outcome_assess_3 |
| Follow up | character (mean, median, max, min) | duration_fup_measure |
|  | integer | duration_fup_units |
|  | integer | value_of_ duration_fup |
| Confounders | Binary | confounders_age |
|  | Binary | confounders_sex |
|  | Binary | confounders_education |
|  | Binary | confounders_income |
|  | Binary | confounders_smoking |
|  | Binary | confounders_alcohol_use |
|  | Binary | confounders_physical_acitivity |
|  | Binary | confounders_bmi |
|  | Binary | confounders_dietary_components |
|  | Binary | confoundeers_hypertension |
|  | Binary | confounders_diabetes |
|  | Binary | confounders_hypercholesterolemia |
|  | Binary | confounders_caloric |
|  | Binary | confounders_other |
| Effect_size | numeric | page_num_effect_size |
|  | character | effect_size_measure |
|  | numeric | effect_size |
|  | numeric | lower |
|  | numeric | upper |
|  | Numeric  (0.95,0.99) | CI_uncertainty_type_value |
|  | Numeric (SD) | nonCI_uncertainty_value |
|  | character (SD, SE) | nonCI_uncertainty_type |
|  | Binary | uncertainty_issue |
|  | Binary | subgroup_analysis |
|  | character | subgroup_analysis_free_text |
|  | Binary | effect_size_multi_location |
|  | character | effect_size_multi_location_specify |
|  | Binary | pooled_cohort |
|  | Binary | dose_response |
|  | character | dose_response_detail |
| Cohort | numeric | cohort_person_years_exp |
|  | numeric | cohort_person_years_unexp |
|  | numeric | cohort_person_years_total |
|  | numeric | cohort_number_events_exp |
|  | numeric | cohort_number_events_unexp |
|  | numeric | cohort_number_events_total |
|  | numeric | cohort_sample_size_exp |
|  | numeric | cohort_sample_size_unexp |
|  | numeric | cohort_sample_size_total |
|  | numeric | cohort_dropout_rate |
|  | character | cohort_dropout_assess |
|  | character | cohort_exposed_def |
|  | character | cohort_exp_unit_rr |
|  | character | cohort_exp_level_rr |
|  | character | cohort_unexp_def |
|  | character | cohort_unexp_unit_rr |
|  | character | cohort_unexp_level_rr |
|  | character | cohort_exp_level_dr |
| Other | character | note_modeler |
|  | character | note_sr |
|  | character | extractor |
| Custom | numeric | custom_exp_means_num |
|  | character | custom_exp_biomarker |
|  | numeric | custom_kilometer |
|  | numeric | custom_exp_level_lower |
|  | numeric | custom_exp_level_upper |
|  | numeric | custom_unexp_level_lower |
|  | numeric | custom_unexp_level_upper |
|  | numeric | custom_prospective_lag |
|  | Binary | custom_age_demographer |
|  | Free text | custom_bmi_menopause_free_text |
|  | Free text | custom_cvd_outcome |
|  | Free text | custom_dm_type |
|  | Free text | custom_dm_defn |
|  | numeric | custom_pmid |
|  | Binary | custom_cvd_rep_high_risk |
|  | Free text | custom_drug_class |

**Section 3.**

**Supplementary Table 3.** Study characteristics extracted from included studies

| **Author** | **Year** | **Study name** | **Population** | **Location** | **Study design** | **Sex** | **Follow-up** | **Age start** | **Age end** | **Exposure assessment** | **Endpoint** | **Disease ascertainment** | **Events** | **Sample size** | **Outcomes** |
| --- | --- | --- | --- | --- | --- | --- | --- | --- | --- | --- | --- | --- | --- | --- | --- |
| Juan Juan | 2017 | NHS | US female registered  nurses | the United States | Prospective cohort study | Female | 26 | 30 | 55 | FFQ | Incidence | Administrative  medical records or disease registries | N= 1,550 | 71,750 | Stroke |
| Juan Juan | 2017 | HPFS | American male health professionals | the United States | Prospective cohort study | Male | 24 | 25 | 42 | FFQ | Incidence | Administrative  medical records or disease registries | N= 908 | 42,823 | Stroke |
| Anna Mizrahi | 2009 | The Finnish Mobile Clinic Health Examination Survey | 30 communities from different parts of Finland | Finland | Prospective cohort study | Both | 24 | 40 | 74 | FFQ | Incidence | Administrative medical records or disease registries | N= 625 | 3,932 | Stroke |
| Thanasis G. Tektonidis | 2015 | The Swedish Mammography Cohort | Women residing in Uppsala and Vastmanland counties in central Sweden | Sweden | Prospective cohort study | Female | 10.4 | 40 | 76 | FFQ | Incidence | Administrative medical records or disease registries | N_stroke_= 1532 | 32,921 | Stroke |
| Yang Hu | 2022 | NHS | US female registered  nurses | the United States | Prospective cohort study | Female | 25.8 | 30 | 55 | FFQ | Incidence | Physician diagnosis | N= 3901 | 74,244 | Ischemic heart disease |
| Yang Hu | 2022 | HPFS | American male health professionals | the United States | Prospective cohort study | Male | 25.8 | 40 | 75 | FFQ | Incidence | Physician diagnosis | N= 4753 | 39,455 | Ischemic heart disease |
| Yang Hu | 2022 | NHS II | US female registered  nurses | the United States | Prospective cohort study | Female | 25.8 | 25 | 42 | FFQ | Incidence | Physician diagnosis | N= 817 | 91,430 | Ischemic heart disease |
| Anne Helnes | 2016 | The Diet, Cancer and Health Cohort (female) | All men and women aged 50 to 64 years, born in Denmark,  living in the greater areas of Aarhus or Copenhagen, and with no previous cancer diagnosis in the Danish Cancer Registry were invited | Denmark | Prospective cohort study | Female | 13.6 | 50 | 64 | FFQ | Incidence | Administrative medical records or disease registries | N= 1676 | 28,950 | Ischemic heart disease |
| Anne Helnes | 2016 | The Diet, Cancer and Health Cohort (male) | All men and women aged 50 to 64 years, born in Denmark,  living in the greater areas of Aarhus or Copenhagen, and  with no previous cancer diagnosis in the Danish Cancer  Registry were invited | Denmark | Prospective cohort study | Male | 13.6 | 50 | 64 | FFQ | Incidence | Administrative medical records or disease registries | N= 653 | 25,921 | Ischemic heart disease |
| Susanne Rautiainen | 2012 | The Swedish Mammography Cohort | Women residing in Uppsala and Vastmanland counties in central Sweden | Sweden | Prospective cohort study | Female | 9.9 | 40 | 76 | FFQ | Incidence | Administrative medical records or disease registries | N=1114 | 32,561 | Ischemic heart disease |
| Nina F. Johnsen | 2015 | The Scandinavian HELGA cohort (female) | Participants were all recruited from the general population in 1992-1998, from Denmark, Norway and Sweden | Norway, Denmark, Sweden | Prospective cohort study | Female | 14.2 | 40 | 63 | Semi-quantitative  FFQ | Mortality | Administrative medical records or disease registries | N_IHD_= 298  N_stroke_= 137 | 80,101 | Ischemic heart disease,  Stroke |
| Nina F. Johnsen | 2015 | The Scandinavian HELGA cohort (male) | Participants were all recruited from the general population in 1992-1998, from Denmark, Norway and Sweden | Norway, Denmark, Sweden | Prospective cohort study | Male | 14.2 | 31 | 64 | Semi-quantitative  FFQ | Mortality | Administrative medical records or disease registries | N_IHD_= 858  N_stroke_= 143 | 39,417 | Ischemic heart disease,  Stroke |
| Sumathi Swaminathan | 2021 | The PURE study | 613 communities in 18 low-income, middle-income, and  high-income countries (HIC) in seven geographical regions: North America and Europe,  South America, the Middle East, south Asia, China, southeast Asia, and Africa. | United Arab  Emirates,  Canada,  Sweden,  Poland,  Argentina,  Chile,  Malasia,  Turkey, Iran,  Occupied  Palestinian  territory,  Brazil, South  Africa,  Columbia,  China, India,  Pakistan,  Bangladesh,  Zimbabwe | Prospective cohort study | Both | 9.5 | 35 | 70 | FFQ | Incidence | Physician diagnosis | N_IHD_= 2931  N_stroke_= 3210 | 137,130 | Ischemic heart disease,  Stroke |
| Lyn M Steffen | 2003 | The ARIC Study | African American and white, men and women residents  from Forsyth County, NC;  Jackson, MS; selected suburbs of | the United States | Prospective cohort study | Both | 11 | 45 | 64 | FFQ | Incidence | Administrative  medical records or disease registries | N_IHD_= 535  N_stroke_= 214 | 11,940 | Ischemic heart disease,  Stroke |
| Jiaomei Yang | 2022 | China Kadoorie Biobank | Paticipants from 10 geographical areas of China (5 urban and 5 rural), covering diverse socioeconomic levels, disease patterns, and risk exposures. | China | Prospective cohort study | Both | 11.2 | 30 | 79 | Semi-quantitative  FFQ | Incidence | Administrative  medical records or disease registries | N_stroke_= 8884  NI_HD_= 15472 | 461,047 | Ischemic heart disease,  Stroke |
| David R Jacobs Jr | 2007 | The Iowa Women’s Health Study | Postmenopausal women | the United States | Prospective cohort study | Female | 17 | 55 | 69 | FFQ | Mortality | Administrative medical records or disease registries | N_IHD_= 233  N_stroke_= 89 | 27,312 | Ischemic heart disease,  Stroke |
| Emily Sonestedt | 2015 | The Malmo Diet and Cancer Study | Living in the city of  Malmo¨ were invited to  participate | Sweden | Prospective cohort study | Both | 14 | 44 | 74 | FFQ | Incidence | Administrative medical records or disease registries | N= 2921 | 26,445 | Stroke,  Ischemic heart disease |
| Alysha S. Thompson | 2022 | UK Biobank | Participants attended 1 of 22 assessment centers located across England, Scotland, and  Wales, where they completed a comprehensive baseline assessment, without prevalent CVD, cancer, or fracture at recruitment | United  Kingdom | Prospective cohort study | Both | 11.6 | 40 | 69 | 24h diet questionnaire | Incidence | Administrative medical records or disease registries | N_IHD_= 3253  N_stroke_= 1640  N_CRC_= 959 | 123,134 | Stroke,  Ischemic heart disease,  Colorectal cancer |
| Xiaosheng He | 2019 | NHS | US female registered  nurses | the United States | Prospective cohort study | Female | 25.8 | 30 | 55 | FFQ | Incidence | Administrative  medical records or disease registries | N= 1902 | 90,869 | Colorectal cancer |
| Xiaosheng He | 2019 | HPFS | American male health professionals | the United States | Prospective cohort study | Male | 25.8 | 40 | 75 | FFQ | Incidence | Administrative  medical records or disease registries | N= 1276 | 47,924 | Colorectal cancer |
| Pirjo Pietinen | 1999 | The ATBC | Male smokers residing in  southwestern Finland | Finland | Prospective cohort study | Male | 8 | 50 | 69 | FFQ | Incidence | Administrative  medical records or disease registries | N= 185 | 27,111 | Colorectal cancer |
| S C Larsson | 2005 | The Swedish Mammography Cohort | Women residing in  Uppsala and Vastmanland counties in central Sweden | Sweden | Prospective cohort study | Female | 14.8 | 40 | 76 | FFQ | Incidence | Administrative medical records or disease registries | N= 805 | 61,433 | Colorectal cancer |
| Guri Skeie | 2013 | The Scandinavian HELGA cohort | Prospectively  diagnosed with CRC were all recruited from the general population in 1992-1998, from Denmark, Norway and Sweden | Norway, Denmark, Sweden | Prospective cohort study | Female | 7 | 40 | 64 | Semi-quantitative  FFQ | Mortality | Administrative medical records or disease registries | N= 197 | 590 | Colorectal cancer |
| Guri Skeie | 2013 | The Scandinavian HELGA cohort | Prospectively  diagnosed with CRC were all recruited from the general population in 1992-1998, from Denmark, Norway and Sweden | Norway, Denmark, Sweden | Prospective cohort study | Male | 7 | 40 | 64 | Semi-quantitative  FFQ | Mortality | Administrative medical records or disease registries | N= 193 | 529 | Colorectal cancer |
| Autumn G Hullings | 2020 | The NIH-AARP Diet and Health Study | US retired civilians | the United states | Prospective cohort study | Both | 16 | 50 | 71 | FFQ | Incidence | Administrative medical records or disease registries | N= 10200 | 478,994 | Colorectal cancer |
| R Egeberg | 2010 | The Diet, Cancer and Health Cohort (female) | All men and women aged 50 to 64 years, born in Denmark,  living in the greater areas of Aarhus or Copenhagen, and with no previous cancer diagnosis in the Danish Cancer  Registry were invited | Denmark | Prospective cohort study | Female | 10.6 | 50 | 64 | FFQ | Incidence | Administrative medical records or disease registries | N= 413 | 29,189 | Colorectal cancer |
| R Egeberg | 2010 | The Diet, Cancer and Health Cohort  (male) | All men and women aged 50 to 64 years, born in Denmark,  living in the greater areas of Aarhus or Copenhagen, and with no previous cancer diagnosis in the Danish Cancer  Registry were invited | Denmark | Prospective cohort study | Male | 10.6 | 50 | 64 | FFQ | Incidence | Administrative medical records or disease registries | N= 331 | 26,630 | Colorectal cancer |
| Caroline Y.Um | 2020 | The CPS-II Nutrition Cohort | Participants were all recruited from the general population in the United states | the United States | Prospective cohort study | Female | 9.8 | 50 | 74 | FFQ | Incidence | Administrative medical records or disease registries | N= 908 | 62,031 | Colorectal cancer |
| Caroline Y.Um | 2020 | The CPS-II Nutrition Cohort | Participants were all recruited from the general population in the United states | the United States | Prospective cohort study | Male | 9.8 | 50 | 74 | FFQ | Incidence | Administrative medical records or disease registries | N= 834 | 50,118 | Colorectal cancer |
| Cecilie Kyre | 2018 | The Diet, Cancer, and Health cohort  (female) | All men and women aged 50 to 64 years, born in Denmark,  living in the greater areas of Aarhus or Copenhagen, and  with no previous cancer diagnosis in the Danish Cancer  Registry were invited | Denmark | Prospective cohort study | Female | 15 | 50 | 64 | FFQ | Incidence | Administrative medical records or disease registries | N= 3301 | 29,214 | Type 2 diabetes |
| Cecilie Kyre | 2018 | The Diet, Cancer, and Health cohort  (male) | All men and women aged 50 to 64 years, born in Denmark,  living in the greater areas of Aarhus or Copenhagen, and  with no previous cancer diagnosis in the Danish Cancer  Registry were invited | Denmark | Prospective cohort study | Male | 15 | 50 | 64 | FFQ | Incidence | Administrative medical records or disease registries | N= 4116 | 26,251 | Type 2 diabetes |
| Katie A Meyer | 2000 | The Iowa Women’s Health Study | US postmenopausal  women | the United States | Prospective cohort study | Female | 6 | 55 | 69 | FFQ | Incidence | Self-report | N= 891 | 35,988 | Type 2 diabetes |
| Jie Li | 2022 | The Women’s Health Initiative | US postmenopausal  women | the United States | Prospective cohort study | Female | 15.8 | 50 | 79 | FFQ | Incidence | Self-report | N= 15,842 | 108,681 | Type 2 diabetes |
| Rob Van Dam | 2006 | The Black Women’s Health Study | Recruited from subscribers to Essence Magazine, members of professional organisations, and friends/relatives of early  respondents | the United States | Prospective cohort study | Female | 8 | 21 | 69 | FFQ | Incidence | Self-report | N= 1964 | 41,186 | Type 2 diabetes |
| Yang Hu | 2020 | NHS | US female registered  nurses | the United States | Prospective cohort study | Female | 24 | 30 | 55 | FFQ | Incidence | Physician diagnosis | N= 2229 | 69,139 | Type 2 diabetes |
| Yang Hu | 2020 | NHS II | US female registered  nurses | the United States | Prospective cohort study | Female | 24 | 25 | 42 | FFQ | Incidence | Physician diagnosis | N= 2156 | 89,130 | Type 2 diabetes |
| Mirkka Maukonen | 2023 | the ATBC | Male smokers residing in  southwestern Finland | Finland | Prospective cohort study | Male | 10.9 | 50 | 70 | FFQ | Incidence | Administrative medical records or disease registries | N= 654 | 25,342 | Type 2 diabetes |
| Mirkka Maukonen | 2023 | The Health 2000 Survey | Populations aged at least 30 years in 2000 from the individual-level population register in order to create a representative sample of the Finnish population | Finland | Prospective cohort study | Both | 15.2 | 30 | 99 | FFQ | Incidence | Administrative medical records or disease registries | N= 608 | 5,695 | Type 2 diabetes |
| Mirkka Maukonen | 2023 | The Helsinki Birth Cohort Study | Subjects born at Helsinki University Central Hospital or at the Midwives’ Hospital in Helsinki, Finland. | Finland | Prospective cohort study | Both | 11.1 | 56 | 69 | FFQ | Incidence | Administrative medical records or disease registries | N= 70 | 1,938 | Type 2 diabetes |
| Mirkka Maukonen | 2023 | The Dietary, Lifestyle, and  Genetic determinants of  Obesity and Metabolic syn_x005f drome 2007 Study | A random sample of 10,000 people aged 25–74 years was drawn from the Finnish population register in five  geographic areas | Finland | Prospective cohort study | Both | 10.8 | 25 | 74 | FFQ | Incidence | Administrative medical records or disease registries | N= 308 | 4,447 | Type 2 diabetes |
| Mirkka Maukonen | 2023 | The National FINRISK 2012  Study | The adult 25 to 74-year-old permanent residents (with at least 1 year of residency and a personal identification code) of Finland | Finland | Prospective cohort study | Both | 5.8 | 25 | 74 | FFQ | Incidence | Administrative medical records or disease registries | N= 110 | 4,240 | Type 2 diabetes |
| Jukka Montonen | 2018 | The Finnish Mobile Clinic Health Examination Survey | 30 communities from different parts of Finland | Finland | Prospective cohort study | Both | 10 | 40 | 69 | FFQ | Incidence | Administrative medical records or disease registries | N= 156 | 4,316 | Type 2 diabetes |
| Tina Wirstrom | 2013 | Swedish middle-aged men and women | Women and men aged 35-56 and without known diabetes were enrolled for a baseline investigation between the years 1992 and 1998 | Sweden | Prospective cohort study | Both | 9 | 35 | 56 | FFQ | Incidence | Physician diagnosis | N= 330 | 5,477 | Type 2 diabetes |
| Yang Hu | 2020 | HPFS | American male health professionals | the United States | Prospective cohort study | Male | 24 | 40 | 75 | FFQ | Incidence | Physician diagnosis | N= 934 | 36,525 | Type 2 diabetes |

**Section 4. Study quality assessment with study characteristics**

Based on BPRF evaluation criteria, we calculated a quality score for each study used in this analysis to provide a broad sense of data quality in **Supplementary Table 4-7**. The overall score assessment was measured from 0 to 5, where 0 indicated the least bias and 5 indicated the most bias.

**Supplementary Table 4.** Study quality for studies about T2D applied in the models

| **Study** | **Outcome** | **cv_exposure_self report (objective-0**  **vs self report-1)** | **cv_outcome_self report**  **(objective-0**  **vs self report-1)** | **cv_exposure_study (multiple-0,**  **single-1)** | **cv_adj**  **(age,sex,calorie,income,education-**  **0; age,sex,calorie-1; age,sex-2)** | **Quality**  **Score**  **(best-0, worst -5)** |
| --- | --- | --- | --- | --- | --- | --- |
| Jukka Montonen, 2018 | Type 2 Diabetes | 1 | 0 | 1 | 0 | 2 |
| Jukka Montonen, 2018 | Type 2 Diabetes | 1 | 0 | 1 | 0 | 2 |
| Jukka Montonen, 2018 | Type 2 Diabetes | 1 | 0 | 1 | 0 | 2 |
| Cecilie Kyrø, 2018 | Type 2 Diabetes | 1 | 0 | 1 | 0 | 2 |
| Cecilie Kyrø, 2018 | Type 2 Diabetes | 1 | 0 | 1 | 0 | 2 |
| Cecilie Kyrø, 2018 | Type 2 Diabetes | 1 | 0 | 1 | 0 | 2 |
| Cecilie Kyrø, 2018 | Type 2 Diabetes | 1 | 0 | 1 | 0 | 2 |
| Cecilie Kyrø, 2018 | Type 2 Diabetes | 1 | 0 | 1 | 0 | 2 |
| Cecilie Kyrø, 2018 | Type 2 Diabetes | 1 | 0 | 1 | 0 | 2 |
| Tina Wirstrom, 2013 | Type 2 Diabetes | 1 | 0 | 1 | 1 | 3 |
| Tina Wirstrom, 2013 | Type 2 Diabetes | 1 | 0 | 1 | 1 | 3 |
| Tina Wirstrom, 2013 | Type 2 Diabetes | 1 | 0 | 1 | 1 | 3 |
| Tina Wirstrom, 2013 | Type 2 Diabetes | 1 | 0 | 1 | 1 | 3 |
| Tina Wirstrom, 2013 | Type 2 Diabetes | 1 | 0 | 1 | 1 | 3 |
| Tina Wirstrom, 2013 | Type 2 Diabetes | 1 | 0 | 1 | 1 | 3 |
| Katie A Meyer, 2000 | Type 2 Diabetes | 1 | 1 | 1 | 1 | 4 |
| Katie A Meyer, 2000 | Type 2 Diabetes | 1 | 1 | 1 | 1 | 4 |
| Katie A Meyer, 2000 | Type 2 Diabetes | 1 | 1 | 1 | 1 | 4 |
| Katie A Meyer, 2000 | Type 2 Diabetes | 1 | 1 | 1 | 1 | 4 |
| Jie Li, 2022 | Type 2 Diabetes | 1 | 1 | 1 | 0 | 3 |
| Jie Li, 2022 | Type 2 Diabetes | 1 | 1 | 1 | 0 | 3 |
| Jie Li, 2022 | Type 2 Diabetes | 1 | 1 | 1 | 0 | 3 |
| Jie Li, 2022 | Type 2 Diabetes | 1 | 1 | 1 | 0 | 3 |
| ROB VAN DAM, 2006 | Type 2 Diabetes | 1 | 1 | 1 | 1 | 4 |
| ROB VAN DAM, 2006 | Type 2 Diabetes | 1 | 1 | 1 | 1 | 4 |
| ROB VAN DAM, 2006 | Type 2 Diabetes | 1 | 1 | 1 | 1 | 4 |
| Yang Hu, 2020 | Type 2 Diabetes | 1 | 0 | 0 | 1 | 2 |
| Yang Hu, 2020 | Type 2 Diabetes | 1 | 0 | 0 | 1 | 2 |
| Yang Hu, 2020 | Type 2 Diabetes | 1 | 0 | 0 | 1 | 2 |
| Yang Hu, 2020 | Type 2 Diabetes | 1 | 0 | 0 | 1 | 2 |
| Yang Hu, 2020 | Type 2 Diabetes | 1 | 0 | 0 | 1 | 2 |
| Yang Hu, 2020 | Type 2 Diabetes | 1 | 0 | 0 | 1 | 2 |
| Yang Hu, 2020 | Type 2 Diabetes | 1 | 0 | 0 | 1 | 2 |
| Yang Hu, 2020 | Type 2 Diabetes | 1 | 0 | 0 | 1 | 2 |
| Yang Hu, 2020 | Type 2 Diabetes | 1 | 0 | 0 | 1 | 2 |
| Yang Hu, 2020 | Type 2 Diabetes | 1 | 0 | 0 | 1 | 2 |
| Yang Hu, 2020 | Type 2 Diabetes | 1 | 0 | 0 | 1 | 2 |
| Yang Hu, 2020 | Type 2 Diabetes | 1 | 0 | 0 | 1 | 2 |
| Mirkka Maukonen, 2023 | Type 2 Diabetes | 1 | 0 | 0 | 2 | 3 |
| Mirkka Maukonen, 2023 | Type 2 Diabetes | 1 | 0 | 0 | 2 | 3 |

**Supplementary Table 5.** Study quality for studies about CRC applied in the models

| **Study** | **Outcome** | **cv_exposure_self report (objective-0**  **vs self report-1)** | **cv_outcome_self report**  **(objective-0**  **vs self report-1)** | **cv_exposure_study (multiple-0,**  **single-1)** | **cv_adj**  **(age, sex, calorie, income, education-0; age, sex, calorie-1; age,sex-2)** | **Quality**  **Score**  **(best-0,**  **worst-5)** |
| --- | --- | --- | --- | --- | --- | --- |
| Xiaosheng He, 2019 | CRC | 1 | 0 | 0 | 1 | 2 |
| Xiaosheng He, 2019 | CRC | 1 | 0 | 0 | 1 | 2 |
| Xiaosheng He, 2019 | CRC | 1 | 0 | 0 | 1 | 2 |
| Xiaosheng He, 2019 | CRC | 1 | 0 | 0 | 1 | 2 |
| Xiaosheng He, 2019 | CRC | 1 | 0 | 0 | 1 | 2 |
| Xiaosheng He, 2019 | CRC | 1 | 0 | 0 | 1 | 2 |
| Xiaosheng He, 2019 | CRC | 1 | 0 | 0 | 1 | 2 |
| Xiaosheng He, 2019 | CRC | 1 | 0 | 0 | 1 | 2 |
| Xiaosheng He, 2019 | CRC | 1 | 0 | 0 | 1 | 2 |
| Xiaosheng He, 2019 | CRC | 1 | 0 | 0 | 1 | 2 |
| Xiaosheng He, 2019 | CRC | 1 | 0 | 0 | 1 | 2 |
| Xiaosheng He, 2019 | CRC | 1 | 0 | 0 | 1 | 2 |
| Xiaosheng He, 2019 | CRC | 1 | 0 | 0 | 1 | 2 |
| Xiaosheng He, 2019 | CRC | 1 | 0 | 0 | 1 | 2 |
| Xiaosheng He, 2019 | CRC | 1 | 0 | 0 | 1 | 2 |
| Xiaosheng He, 2019 | CRC | 1 | 0 | 0 | 1 | 2 |
| Xiaosheng He, 2019 | CRC | 1 | 0 | 0 | 1 | 2 |
| Xiaosheng He, 2019 | CRC | 1 | 0 | 0 | 1 | 2 |
| Pirjo Pietinen, 1999 | CRC | 1 | 0 | 1 | 2 | 4 |
| Pirjo Pietinen, 1999 | CRC | 1 | 0 | 1 | 2 | 4 |
| Pirjo Pietinen, 1999 | CRC | 1 | 0 | 1 | 2 | 4 |
| Caroline Y.Um, 2020 | CRC | 1 | 0 | 0 | 0 | 1 |
| Caroline Y.Um, 2020 | CRC | 1 | 0 | 0 | 0 | 1 |
| Caroline Y.Um, 2020 | CRC | 1 | 0 | 0 | 0 | 1 |
| Caroline Y.Um, 2020 | CRC | 1 | 0 | 0 | 0 | 1 |
| Caroline Y.Um, 2020 | CRC | 1 | 0 | 0 | 0 | 1 |
| Caroline Y.Um, 2020 | CRC | 1 | 0 | 0 | 0 | 1 |
| Caroline Y.Um, 2020 | CRC | 1 | 0 | 0 | 0 | 1 |
| Caroline Y.Um, 2020 | CRC | 1 | 0 | 0 | 0 | 1 |
| Guri Skeie, 2013 | CRC | 1 | 0 | 1 | 0 | 2 |
| Guri Skeie, 2013 | CRC | 1 | 0 | 1 | 0 | 2 |
| Guri Skeie, 2013 | CRC | 1 | 0 | 1 | 0 | 2 |
| Guri Skeie, 2013 | CRC | 1 | 0 | 1 | 0 | 2 |
| S C Larsson, 2005 | CRC | 1 | 0 | 1 | 0 | 2 |
| S C Larsson, 2005 | CRC | 1 | 0 | 1 | 0 | 2 |
| S C Larsson, 2005 | CRC | 1 | 0 | 1 | 0 | 2 |
| S C Larsson, 2005 | CRC | 1 | 0 | 1 | 0 | 2 |
| Autumn G Hullings, 2020 | CRC | 1 | 0 | 0 | 0 | 1 |
| Autumn G Hullings, 2020 | CRC | 1 | 0 | 0 | 0 | 1 |
| Autumn G Hullings, 2020 | CRC | 1 | 0 | 0 | 0 | 1 |
| Autumn G Hullings, 2020 | CRC | 1 | 0 | 0 | 0 | 1 |
| Alysha S. Thompson, 2022 | CRC | 1 | 0 | 0 | 0 | 1 |
| Alysha S. Thompson, 2022 | CRC | 1 | 0 | 0 | 0 | 1 |
| Alysha S. Thompson, 2022 | CRC | 1 | 0 | 0 | 0 | 1 |
| R Egeberg, 2010 | CRC | 1 | 0 | 1 | 2 | 4 |
| R Egeberg, 2010 | CRC | 1 | 0 | 1 | 2 | 4 |
| R Egeberg, 2010 | CRC | 1 | 0 | 1 | 2 | 4 |
| R Egeberg, 2010 | CRC | 1 | 0 | 1 | 2 | 4 |
| R Egeberg, 2010 | CRC | 1 | 0 | 1 | 2 | 4 |
| R Egeberg, 2010 | CRC | 1 | 0 | 1 | 2 | 4 |
| R Egeberg, 2010 | CRC | 1 | 0 | 1 | 2 | 4 |
| R Egeberg, 2010 | CRC | 1 | 0 | 1 | 2 | 4 |
| R Egeberg, 2010 | CRC | 1 | 0 | 1 | 2 | 4 |
| R Egeberg, 2010 | CRC | 1 | 0 | 1 | 2 | 4 |
| R Egeberg, 2010 | CRC | 1 | 0 | 1 | 2 | 4 |
| R Egeberg, 2010 | CRC | 1 | 0 | 1 | 2 | 4 |

**Supplementary Table 6.** Study quality for studies about IHD applied in the models

| **Study** | **Outcome** | **cv_exposure_self report (objective-0**  **vs self report-1)** | **cv_outcome_self report**  **(objective-0**  **vs self report-1)** | **cv_exposure_study (multiple-0,**  **single-1)** | **cv_adj**  **(age,sex,calorie,income,education-**  **0; age,sex,calorie-1; age,sex-2)** | **Quality**  **Score**  **(best-0,**  **worst-5)** |
| --- | --- | --- | --- | --- | --- | --- |
| Yang Hu, 2022 | IHD | 1 | 0 | 0 | 1 | 2 |
| Yang Hu, 2022 | IHD | 1 | 0 | 0 | 1 | 2 |
| Yang Hu, 2022 | IHD | 1 | 0 | 0 | 1 | 2 |
| Yang Hu, 2022 | IHD | 1 | 0 | 0 | 1 | 2 |
| Yang Hu, 2022 | IHD | 1 | 0 | 0 | 1 | 2 |
| Yang Hu, 2022 | IHD | 1 | 0 | 0 | 1 | 2 |
| Yang Hu, 2022 | IHD | 1 | 0 | 0 | 1 | 2 |
| Yang Hu, 2022 | IHD | 1 | 0 | 0 | 1 | 2 |
| Yang Hu, 2022 | IHD | 1 | 0 | 0 | 1 | 2 |
| Yang Hu, 2022 | IHD | 1 | 0 | 0 | 1 | 2 |
| Yang Hu, 2022 | IHD | 1 | 0 | 0 | 1 | 2 |
| Yang Hu, 2022 | IHD | 1 | 0 | 0 | 1 | 2 |
| David R Jacobs Jr, 2007 | IHD | 1 | 0 | 1 | 1 | 3 |
| David R Jacobs Jr, 2007 | IHD | 1 | 0 | 1 | 1 | 3 |
| David R Jacobs Jr, 2007 | IHD | 1 | 0 | 1 | 1 | 3 |
| David R Jacobs Jr, 2007 | IHD | 1 | 0 | 1 | 1 | 3 |
| Nina F. Johnsen, 2015 | IHD | 1 | 0 | 1 | 0 | 2 |
| Nina F. Johnsen, 2015 | IHD | 1 | 0 | 1 | 0 | 2 |
| Nina F. Johnsen, 2015 | IHD | 1 | 0 | 1 | 0 | 2 |
| Nina F. Johnsen, 2015 | IHD | 1 | 0 | 1 | 0 | 2 |
| Nina F. Johnsen, 2015 | IHD | 1 | 0 | 1 | 0 | 2 |
| Nina F. Johnsen, 2015 | IHD | 1 | 0 | 1 | 0 | 2 |
| Jiaomei Yang, 2022 | IHD | 1 | 0 | 0 | 1 | 2 |
| Jiaomei Yang, 2022 | IHD | 1 | 0 | 0 | 1 | 2 |
| Jiaomei Yang, 2022 | IHD | 1 | 0 | 0 | 1 | 2 |
| Susanne Rautiainen, 2012 | IHD | 1 | 0 | 1 | 0 | 2 |
| Susanne Rautiainen, 2012 | IHD | 1 | 0 | 1 | 0 | 2 |
| Susanne Rautiainen, 2012 | IHD | 1 | 0 | 1 | 0 | 2 |
| Anne Helnæs, 2016 | IHD | 1 | 0 | 1 | 1 | 3 |
| Anne Helnæs, 2016 | IHD | 1 | 0 | 1 | 1 | 3 |
| Anne Helnæs, 2016 | IHD | 1 | 0 | 1 | 1 | 3 |
| Anne Helnæs, 2016 | IHD | 1 | 0 | 1 | 1 | 3 |
| Anne Helnæs, 2016 | IHD | 1 | 0 | 1 | 1 | 3 |
| Anne Helnæs, 2016 | IHD | 1 | 0 | 1 | 1 | 3 |
| Sumathi Swaminathan, 2021 | IHD | 1 | 0 | 1 | 0 | 2 |
| Sumathi Swaminathan, 2021 | IHD | 1 | 0 | 1 | 0 | 2 |
| Sumathi Swaminathan, 2021 | IHD | 1 | 0 | 1 | 0 | 2 |
| Lyn M Steffen, 2003 | IHD | 1 | 0 | 0 | 0 | 1 |
| Lyn M Steffen, 2003 | IHD | 1 | 0 | 0 | 0 | 1 |
| Lyn M Steffen, 2003 | IHD | 1 | 0 | 0 | 0 | 1 |
| Lyn M Steffen, 2003 | IHD | 1 | 0 | 0 | 0 | 1 |
| Alysha S. Thompson, 2022 | IHD | 1 | 0 | 0 | 0 | 1 |
| Alysha S. Thompson, 2022 | IHD | 1 | 0 | 0 | 0 | 1 |
| Alysha S. Thompson, 2022 | IHD | 1 | 0 | 0 | 0 | 1 |
| Emily Sonestedt, 2015 | IHD | 1 | 0 | 1 | 0 | 2 |
| Emily Sonestedt, 2015 | IHD | 1 | 0 | 1 | 0 | 2 |
| Emily Sonestedt, 2015 | IHD | 1 | 0 | 1 | 0 | 2 |
| Emily Sonestedt, 2015 | IHD | 1 | 0 | 1 | 0 | 2 |

**Supplementary Table 7.** Study quality for studies about stroke applied in the models

| **Study** | **Outcome** | **cv_exposure_self report (objective-0**  **vs self report-1)** | **cv_outcome_self report**  **(objective-0**  **vs self report-1)** | **cv_exposure_study (multiple-0,**  **single-1)** | **cv_adj**  **(age,sex,calorie,income,education-0; age,sex,calorie-1; age,sex-2)** | **Quality**  **Score**  **(best-0,**  **worst-5)** |
| --- | --- | --- | --- | --- | --- | --- |
| Nina F. Johnsen, 2015 | Stroke | 1 | 0 | 1 | 0 | 2 |
| Nina F. Johnsen, 2015 | Stroke | 1 | 0 | 1 | 0 | 2 |
| Nina F. Johnsen, 2015 | Stroke | 1 | 0 | 1 | 0 | 2 |
| Nina F. Johnsen, 2015 | Stroke | 1 | 0 | 1 | 0 | 2 |
| Nina F. Johnsen, 2015 | Stroke | 1 | 0 | 1 | 0 | 2 |
| Nina F. Johnsen, 2015 | Stroke | 1 | 0 | 1 | 0 | 2 |
| Juan Juan, 2017 | Stroke | 1 | 0 | 0 | 0 | 1 |
| Juan Juan, 2017 | Stroke | 1 | 0 | 0 | 0 | 1 |
| Juan Juan, 2017 | Stroke | 1 | 0 | 0 | 0 | 1 |
| Juan Juan, 2017 | Stroke | 1 | 0 | 0 | 0 | 1 |
| Juan Juan, 2017 | Stroke | 1 | 0 | 0 | 0 | 1 |
| Juan Juan, 2017 | Stroke | 1 | 0 | 0 | 0 | 1 |
| Juan Juan, 2017 | Stroke | 1 | 0 | 0 | 0 | 1 |
| Juan Juan, 2017 | Stroke | 1 | 0 | 0 | 0 | 1 |
| Lyn M Steffen, 2003 | Stroke | 1 | 0 | 0 | 0 | 1 |
| Lyn M Steffen, 2003 | Stroke | 1 | 0 | 0 | 0 | 1 |
| Lyn M Steffen, 2003 | Stroke | 1 | 0 | 0 | 0 | 1 |
| Lyn M Steffen, 2003 | Stroke | 1 | 0 | 0 | 0 | 1 |
| Thanasis G. Tektonidis, 2015 | Stroke | 1 | 0 | 1 | 2 | 4 |
| Thanasis G. Tektonidis, 2015 | Stroke | 1 | 0 | 1 | 2 | 4 |
| Thanasis G. Tektonidis, 2015 | Stroke | 1 | 0 | 1 | 2 | 4 |
| Thanasis G. Tektonidis, 2015 | Stroke | 1 | 0 | 1 | 2 | 4 |
| Thanasis G. Tektonidis, 2015 | Stroke | 1 | 0 | 1 | 2 | 4 |
| Thanasis G. Tektonidis, 2015 | Stroke | 1 | 0 | 1 | 2 | 4 |
| Jiaomei Yang, 2022 | Stroke | 1 | 0 | 0 | 1 | 2 |
| Jiaomei Yang, 2022 | Stroke | 1 | 0 | 0 | 1 | 2 |
| Jiaomei Yang, 2022 | Stroke | 1 | 0 | 0 | 1 | 2 |
| Jiaomei Yang, 2022 | Stroke | 1 | 0 | 0 | 1 | 2 |
| Jiaomei Yang, 2022 | Stroke | 1 | 0 | 0 | 1 | 2 |
| Jiaomei Yang, 2022 | Stroke | 1 | 0 | 0 | 1 | 2 |
| David R Jacobs Jr, 2007 | Stroke | 1 | 0 | 1 | 1 | 3 |
| David R Jacobs Jr, 2007 | Stroke | 1 | 0 | 1 | 1 | 3 |
| David R Jacobs Jr, 2007 | Stroke | 1 | 0 | 1 | 1 | 3 |
| David R Jacobs Jr, 2007 | Stroke | 1 | 0 | 1 | 1 | 3 |
| Anna Mizrahi, 2009 | Stroke | 1 | 0 | 1 | 0 | 2 |
| Anna Mizrahi, 2009 | Stroke | 1 | 0 | 1 | 0 | 2 |
| Anna Mizrahi, 2009 | Stroke | 1 | 0 | 1 | 0 | 2 |
| Alysha S. Thompson, 2022 | Stroke | 1 | 0 | 0 | 0 | 1 |
| Alysha S. Thompson, 2022 | Stroke | 1 | 0 | 0 | 0 | 1 |
| Alysha S. Thompson, 2022 | Stroke | 1 | 0 | 0 | 0 | 1 |
| Alysha S. Thompson, 2022 | Stroke | 1 | 0 | 0 | 0 | 1 |
| Alysha S. Thompson, 2022 | Stroke | 1 | 0 | 0 | 0 | 1 |
| Alysha S. Thompson, 2022 | Stroke | 1 | 0 | 0 | 0 | 1 |
| Emily Sonestedt, 2015 | Stroke | 1 | 0 | 1 | 0 | 2 |
| Emily Sonestedt, 2015 | Stroke | 1 | 0 | 1 | 0 | 2 |
| Emily Sonestedt, 2015 | Stroke | 1 | 0 | 1 | 0 | 2 |
| Emily Sonestedt, 2015 | Stroke | 1 | 0 | 1 | 0 | 2 |
| Sumathi Swaminathan, 2021 | Stroke | 1 | 1 | 1 | 0 | 3 |
| Sumathi Swaminathan, 2021 | Stroke | 1 | 1 | 1 | 0 | 3 |
| Sumathi Swaminathan, 2021 | Stroke | 1 | 1 | 1 | 0 | 3 |

**Section 5.**

The risk-outcome pairs, reference group exposure, alternative group exposure, log effect size, and log effect size (standard deviation, sd) identified from each publications are provided in **Supplementary Table 8**.

**Supplementary Table 8.** Summary results from included studies for formal analysis

| **Study** | **Outcome** | **ref_exposure** | **alt_exposure** | **ln_effect** | **ln_se** |
| --- | --- | --- | --- | --- | --- |
| Xiaosheng He, 2019 | CRC | 0-5g | 5-8g | -0.01 | 0.12 |
| Xiaosheng He, 2019 | CRC | 0-5g | 8-10g | -0.01 | 0.12 |
| Xiaosheng He, 2019 | CRC | 0-5g | 10-13g | 0.13 | 0.11 |
| Xiaosheng He, 2019 | CRC | 0-5g | 13-15g | 0.02 | 0.12 |
| Xiaosheng He, 2019 | CRC | 0-5g | 15-18g | -0.07 | 0.12 |
| Xiaosheng He, 2019 | CRC | 0-5g | 18-21g | -0.02 | 0.12 |
| Xiaosheng He, 2019 | CRC | 0-5g | 21-25g | -0.07 | 0.12 |
| Xiaosheng He, 2019 | CRC | 0-5g | 25-34g | 0.01 | 0.12 |
| Xiaosheng He, 2019 | CRC | 0-5g | 34-46g | 0.08 | 0.13 |
| Xiaosheng He, 2019 | CRC | 0-7g | 7-12g | 0.11 | 0.12 |
| Xiaosheng He, 2019 | CRC | 0-7g | 12-16g | -0.06 | 0.12 |
| Xiaosheng He, 2019 | CRC | 0-7g | 16-20g | -0.08 | 0.13 |
| Xiaosheng He, 2019 | CRC | 0-7g | 20-23g | 0.12 | 0.12 |
| Xiaosheng He, 2019 | CRC | 0-7g | 23-28g | -0.03 | 0.13 |
| Xiaosheng He, 2019 | CRC | 0-7g | 28-33g | -0.15 | 0.13 |
| Xiaosheng He, 2019 | CRC | 0-7g | 33-39g | -0.09 | 0.13 |
| Xiaosheng He, 2019 | CRC | 0-7g | 39-51g | -0.04 | 0.13 |
| Xiaosheng He, 2019 | CRC | 0-7g | 51-73g | -0.31 | 0.14 |
| Pirjo Pietinen, 1999 | CRC | 0-139g | 139-219g | 0.18 | 0.22 |
| Pirjo Pietinen, 1999 | CRC | 0-139g | 219-315g | 0.18 | 0.21 |
| Pirjo Pietinen, 1999 | CRC | 0-139g | 315-374g | 0.00 | 0.21 |
| Caroline Y.Um, 2020 | CRC | 0-19g | 19-37g | -0.04 | 0.11 |
| Caroline Y.Um, 2020 | CRC | 0-19g | 37-64g | -0.17 | 0.11 |
| Caroline Y.Um, 2020 | CRC | 0-19g | 64-117g | -0.12 | 0.11 |
| Caroline Y.Um, 2020 | CRC | 0-19g | 117-168g | -0.26 | 0.12 |
| Caroline Y.Um, 2020 | CRC | 0-20g | 20-37g | -0.06 | 0.11 |
| Caroline Y.Um, 2020 | CRC | 0-20g | 37-63g | -0.05 | 0.11 |
| Caroline Y.Um, 2020 | CRC | 0-20g | 63-117g | 0.10 | 0.11 |
| Caroline Y.Um, 2020 | CRC | 0-20g | 117-174g | 0.10 | 0.11 |
| Guri Skeie, 2013 | CRC | 0-108g | 108-166g | -0.04 | 0.19 |
| Guri Skeie, 2013 | CRC | 0-108g | 166-196g | 0.06 | 0.20 |
| Guri Skeie, 2013 | CRC | 0-93g | 93-157g | 0.23 | 0.18 |
| Guri Skeie, 2013 | CRC | 0-93g | 157-195g | 0.10 | 0.20 |
| S C Larsson, 2005 | CRC | 0-45g | 45-72g | 0.00 | 0.11 |
| S C Larsson, 2005 | CRC | 0-45g | 75-102g | 0.05 | 0.11 |
| S C Larsson, 2005 | CRC | 0-45g | 105-132g | -0.04 | 0.13 |
| S C Larsson, 2005 | CRC | 0-45g | 135-150g | -0.22 | 0.15 |
| Autumn G Hullings, 2020 | CRC | 0-10g | 10-18g | -0.06 | 0.03 |
| Autumn G Hullings, 2020 | CRC | 0-10g | 18-28g | -0.08 | 0.03 |
| Autumn G Hullings, 2020 | CRC | 0-10g | 28-48g | -0.13 | 0.03 |
| Autumn G Hullings, 2020 | CRC | 0-10g | 48-56g | -0.17 | 0.04 |
| Alysha S. Thompson, 2022 | CRC | 0-54g | 57-65g | 0.01 | 0.09 |
| Alysha S. Thompson, 2022 | CRC | 0-54g | 68-77g | -0.08 | 0.09 |
| Alysha S. Thompson, 2022 | CRC | 0-54g | 80-174g | -0.14 | 0.10 |
| R Egeberg, 2010 | CRC | 0-75g | 75-115g | -0.05 | 0.18 |
| R Egeberg, 2010 | CRC | 0-75g | 115-160g | -0.36 | 0.20 |
| R Egeberg, 2010 | CRC | 0-75g | 160-243g | -0.49 | 0.18 |
| R Egeberg, 2010 | CRC | 0-75g | 75-115g | 0.37 | 0.23 |
| R Egeberg, 2010 | CRC | 0-75g | 115-160g | -0.17 | 0.26 |
| R Egeberg, 2010 | CRC | 0-75g | 160-241g | -0.13 | 0.22 |
| R Egeberg, 2010 | CRC | 0-75g | 75-115g | 0.08 | 0.19 |
| R Egeberg, 2010 | CRC | 0-75g | 115-160g | 0.12 | 0.20 |
| R Egeberg, 2010 | CRC | 0-75g | 160-220g | -0.08 | 0.19 |
| R Egeberg, 2010 | CRC | 0-75g | 75-115g | -0.36 | 0.25 |
| R Egeberg, 2010 | CRC | 0-75g | 115-160g | -0.62 | 0.30 |
| R Egeberg, 2010 | CRC | 0-75g | 160-282g | -0.21 | 0.24 |
| Jukka Montonen 2018 | T2D | 0-109g | 110-162g | 0.05 | 0.20 |
| Jukka Montonen 2018 | T2D | 0-109g | 163-237g | -0.65 | 0.27 |
| Jukka Montonen 2018 | T2D | 0-109g | 238-302g | -0.43 | 0.30 |
| Cecilie Kyrø, 2018 | T2D | 0-94g | 94-130g | -0.06 | 0.04 |
| Cecilie Kyrø, 2018 | T2D | 0-94g | 130-184g | -0.12 | 0.04 |
| Cecilie Kyrø, 2018 | T2D | 0-94g | 184-267g | -0.22 | 0.05 |
| Cecilie Kyrø, 2018 | T2D | 0-70g | 70-111g | -0.08 | 0.05 |
| Cecilie Kyrø, 2018 | T2D | 0-70g | 111-163g | 0.00 | 0.05 |
| Cecilie Kyrø, 2018 | T2D | 0-70g | 163-221g | -0.16 | 0.05 |
| Tina Wirstrom, 2013 | T2D | 0-31g | 31-59g | -0.45 | 0.21 |
| Tina Wirstrom, 2013 | T2D | 0-31g | 59-89g | -0.34 | 0.20 |
| Tina Wirstrom, 2013 | T2D | 0-31g | 31-59g | -0.43 | 0.35 |
| Tina Wirstrom, 2013 | T2D | 0-31g | 59-89g | -0.39 | 0.34 |
| Tina Wirstrom, 2013 | T2D | 0-31g | 31-59g | -0.46 | 0.26 |
| Tina Wirstrom, 2013 | T2D | 0-31g | 59-89g | -0.33 | 0.25 |
| Katie A Meyer, 2000 | T2D | 0-13g | 13-24g | -0.01 | 0.09 |
| Katie A Meyer, 2000 | T2D | 0-13g | 26-34g | -0.02 | 0.10 |
| Katie A Meyer, 2000 | T2D | 0-13g | 36-75g | -0.08 | 0.10 |
| Katie A Meyer, 2000 | T2D | 0-13g | 75-99g | -0.24 | 0.10 |
| Jie Li, 2022 | T2D | 3-10g | 14-20g | -0.05 | 0.03 |
| Jie Li, 2022 | T2D | 0-6g | 25-32g | -0.04 | 0.03 |
| Jie Li, 2022 | T2D | 0-6g | 37-47g | -0.05 | 0.03 |
| Jie Li, 2022 | T2D | 0-6g | 50-107g | -0.08 | 0.05 |
| ROB VAN DAM, 2006 | T2D | 0-4g | 4-41g | -0.17 | 0.05 |
| ROB VAN DAM, 2006 | T2D | 0-4g | 41-54g | -0.27 | 0.08 |
| ROB VAN DAM, 2006 | T2D | 0-4g | 54-101g | -0.37 | 0.07 |
| Yang Hu, 2020 | T2D | 1-3g | 5-7g | -0.15 | 0.03 |
| Yang Hu, 2020 | T2D | 1-3g | 9-12g | -0.22 | 0.03 |
| Yang Hu, 2020 | T2D | 1-3g | 15-19g | -0.34 | 0.04 |
| Yang Hu, 2020 | T2D | 1-3g | 25-39g | -0.39 | 0.04 |
| Yang Hu, 2020 | T2D | 3-7g | 10-12g | -0.11 | 0.03 |
| Yang Hu, 2020 | T2D | 3-7g | 15-19g | -0.17 | 0.04 |
| Yang Hu, 2020 | T2D | 3-7g | 23-28g | -0.27 | 0.04 |
| Yang Hu, 2020 | T2D | 3-7g | 35-49g | -0.31 | 0.04 |
| Yang Hu, 2020 | T2D | 2-5g | 8-12g | -0.17 | 0.05 |
| Yang Hu, 2020 | T2D | 2-5g | 15-19g | -0.21 | 0.05 |
| Yang Hu, 2020 | T2D | 2-5g | 24-30g | -0.33 | 0.05 |
| Yang Hu, 2020 | T2D | 2-5g | 39-58g | -0.33 | 0.06 |
| Mirkka Maukonen, 2023 | T2D | 53-123g | 156-197g | -0.01 | 0.09 |
| Mirkka Maukonen, 2023 | T2D | 53-123g | 229-375g | -0.03 | 0.13 |
| Nina F. Johnsen, 2015 | Stroke | 0-63g | 64-102g | -0.25 | 0.05 |
| Nina F. Johnsen, 2015 | Stroke | 0-63g | 103-128g | -0.26 | 0.05 |
| Nina F. Johnsen, 2015 | Stroke | 0-63g | 129-207g | -0.39 | 0.05 |
| Nina F. Johnsen, 2015 | Stroke | 0-64g | 65-108g | -0.14 | 0.04 |
| Nina F. Johnsen, 2015 | Stroke | 0-64g | 109-148g | -0.30 | 0.04 |
| Nina F. Johnsen, 2015 | Stroke | 0-64g | 149-252g | -0.29 | 0.04 |
| Juan Juan, 2017 | Stroke | 0-7g | 7-13g | -0.19 | 0.09 |
| Juan Juan, 2017 | Stroke | 0-7g | 13-18g | -0.02 | 0.08 |
| Juan Juan, 2017 | Stroke | 0-7g | 18-27g | -0.15 | 0.09 |
| Juan Juan, 2017 | Stroke | 0-7g | 27-48g | 0.03 | 0.08 |
| Juan Juan, 2017 | Stroke | 0-10g | 10-18g | 0.24 | 0.10 |
| Juan Juan, 2017 | Stroke | 0-10g | 18-27g | -0.05 | 0.11 |
| Juan Juan, 2017 | Stroke | 0-10g | 27-40g | -0.19 | 0.11 |
| Juan Juan, 2017 | Stroke | 0-10g | 40-72g | 0.06 | 0.11 |
| Lyn M Steffen, 2003 | Stroke | 0-6g | 9-21g | 0.10 | 0.20 |
| Lyn M Steffen, 2003 | Stroke | 0-6g | 24-33g | -0.24 | 0.23 |
| Lyn M Steffen, 2003 | Stroke | 0-6g | 36-57g | -0.12 | 0.23 |
| Lyn M Steffen, 2003 | Stroke | 0-6g | 60-135g | -0.29 | 0.25 |
| Thanasis G. Tektonidis, 2015 | Stroke | 0-87g | 90-105g | -0.06 | 0.07 |
| Thanasis G. Tektonidis, 2015 | Stroke | 0-87g | 108-119g | -0.12 | 0.08 |
| Thanasis G. Tektonidis, 2015 | Stroke | 0-87g | 119-178g | -0.25 | 0.09 |
| Thanasis G. Tektonidis, 2015 | Stroke | 0-87g | 90-105g | -0.06 | 0.17 |
| Thanasis G. Tektonidis, 2015 | Stroke | 0-87g | 108-119g | -0.16 | 0.18 |
| Thanasis G. Tektonidis, 2015 | Stroke | 0-87g | 119-178g | -0.13 | 0.19 |
| Jiaomei Yang, 2022 | Stroke | 0-11g | 11-18g | -0.02 | 0.01 |
| Jiaomei Yang, 2022 | Stroke | 0-11g | 18-38g | -0.03 | 0.02 |
| Jiaomei Yang, 2022 | Stroke | 0-11g | 38-111g | -0.15 | 0.04 |
| Jiaomei Yang, 2022 | Stroke | 0-11g | 11-18g | -0.06 | 0.02 |
| Jiaomei Yang, 2022 | Stroke | 0-11g | 18-38g | -0.01 | 0.04 |
| Jiaomei Yang, 2022 | Stroke | 0-11g | 38-111g | -0.04 | 0.12 |
| David R Jacobs Jr, 2007 | Stroke | 0-15g | 17-30g | -0.09 | 0.16 |
| David R Jacobs Jr, 2007 | Stroke | 0-15g | 32-45g | -0.17 | 0.16 |
| David R Jacobs Jr, 2007 | Stroke | 0-15g | 47-79g | -0.13 | 0.16 |
| David R Jacobs Jr, 2007 | Stroke | 0-15g | 81-110g | -0.16 | 0.18 |
| Anna Mizrahi, 2009 | Stroke | 0-139g | 140-201g | -0.02 | 0.12 |
| Anna Mizrahi, 2009 | Stroke | 0-139g | 202-279g | 0.17 | 0.12 |
| Anna Mizrahi, 2009 | Stroke | 0-139g | 280-420g | 0.11 | 0.13 |
| Alysha S. Thompson, 2022 | Stroke | 0-54g | 57-65g | -0.04 | 0.08 |
| Alysha S. Thompson, 2022 | Stroke | 0-54g | 68-77g | -0.16 | 0.08 |
| Alysha S. Thompson, 2022 | Stroke | 0-54g | 80-174g | -0.17 | 0.08 |
| Alysha S. Thompson, 2022 | Stroke | 0-54g | 57-65g | 0.06 | 0.13 |
| Alysha S. Thompson, 2022 | Stroke | 0-54g | 68-77g | -0.02 | 0.13 |
| Alysha S. Thompson, 2022 | Stroke | 0-54g | 80-174g | 0.06 | 0.13 |
| Emily Sonestedt, 2015 | Stroke | 0-0g | 0-9g | -0.04 | 0.09 |
| Emily Sonestedt, 2015 | Stroke | 0-0g | 9-21g | -0.08 | 0.09 |
| Emily Sonestedt, 2015 | Stroke | 0-0g | 21-36g | -0.29 | 0.10 |
| Emily Sonestedt, 2015 | Stroke | 0-0g | 36-75g | -0.16 | 0.09 |
| Sumathi Swaminathan, 2021 | Stroke | 154-362g | 0-0g | 0.06 | 0.06 |
| Sumathi Swaminathan, 2021 | Stroke | 154-362g | 9-31g | 0.00 | 0.06 |
| Sumathi Swaminathan, 2021 | Stroke | 154-362g | 62-84g | 0.01 | 0.07 |
| Yang Hu, 2022 | IHD | 1-3g | 5-7g | -0.13 | 0.05 |
| Yang Hu, 2022 | IHD | 1-3g | 9-12g | -0.17 | 0.05 |
| Yang Hu, 2022 | IHD | 1-3g | 15-19g | -0.24 | 0.05 |
| Yang Hu, 2022 | IHD | 1-3g | 25-39g | -0.24 | 0.05 |
| Yang Hu, 2022 | IHD | 3-7g | 10-12g | -0.07 | 0.10 |
| Yang Hu, 2022 | IHD | 3-7g | 15-19g | -0.26 | 0.10 |
| Yang Hu, 2022 | IHD | 3-7g | 23-28g | -0.48 | 0.12 |
| Yang Hu, 2022 | IHD | 3-7g | 35-49g | -0.46 | 0.12 |
| Yang Hu, 2022 | IHD | 2-5g | 8-12g | -0.17 | 0.04 |
| Yang Hu, 2022 | IHD | 2-5g | 15-19g | -0.29 | 0.04 |
| Yang Hu, 2022 | IHD | 2-5g | 24-30g | -0.37 | 0.05 |
| Yang Hu, 2022 | IHD | 2-5g | 39-58g | -0.42 | 0.05 |
| David R Jacobs Jr, 2007 | IHD | 0-15g | 17-30g | 0.01 | 0.10 |
| David R Jacobs Jr, 2007 | IHD | 0-15g | 32-45g | -0.16 | 0.10 |
| David R Jacobs Jr, 2007 | IHD | 0-15g | 47-79g | -0.24 | 0.11 |
| David R Jacobs Jr, 2007 | IHD | 0-15g | 79-110g | -0.33 | 0.12 |
| Nina F. Johnsen, 2015 | IHD | 13-27g | 27-42g | -0.33 | 0.15 |
| Nina F. Johnsen, 2015 | IHD | 13-27g | 42-61g | -0.45 | 0.17 |
| Nina F. Johnsen, 2015 | IHD | 13-27g | 61-95g | -0.58 | 0.17 |
| Nina F. Johnsen, 2015 | IHD | 13-29g | 29-45g | -0.06 | 0.10 |
| Nina F. Johnsen, 2015 | IHD | 13-29g | 45-68g | -0.22 | 0.10 |
| Nina F. Johnsen, 2015 | IHD | 13-29g | 68-111g | -0.16 | 0.10 |
| Jiaomei Yang, 2022 | IHD | 0-11g | 11-18g | 0.07 | 0.05 |
| Jiaomei Yang, 2022 | IHD | 0-11g | 18-38g | 0.09 | 0.06 |
| Jiaomei Yang, 2022 | IHD | 0-11g | 38-111g | -0.05 | 0.08 |
| Susanne Rautiainen, 2012 | IHD | 0-69g | 69-102g | -0.05 | 0.08 |
| Susanne Rautiainen, 2012 | IHD | 0-69g | 102-141g | -0.13 | 0.09 |
| Susanne Rautiainen, 2012 | IHD | 0-69g | 141-174g | -0.12 | 0.09 |
| Anne Helnæs, 2016 | IHD | 10-30g | 30-45g | -0.14 | 0.07 |
| Anne Helnæs, 2016 | IHD | 10-30g | 45-63g | -0.07 | 0.07 |
| Anne Helnæs, 2016 | IHD | 10-30g | 63-87g | -0.13 | 0.08 |
| Anne Helnæs, 2016 | IHD | 9-25g | 25-36g | -0.17 | 0.11 |
| Anne Helnæs, 2016 | IHD | 9-25g | 36-53g | -0.24 | 0.11 |
| Anne Helnæs, 2016 | IHD | 9-25g | 53-87g | -0.27 | 0.12 |
| Sumathi Swaminathan, 2021 | IHD | 154-362g | 0-0g | 0.12 | 0.08 |
| Sumathi Swaminathan, 2021 | IHD | 154-362g | 9-31g | 0.08 | 0.07 |
| Sumathi Swaminathan, 2021 | IHD | 154-362g | 62-84g | 0.13 | 0.07 |
| Lyn M Steffen, 2003 | IHD | 0-6g | 9-21g | -0.27 | 0.14 |
| Lyn M Steffen, 2003 | IHD | 0-6g | 24-33g | -0.07 | 0.13 |
| Lyn M Steffen, 2003 | IHD | 0-6g | 36-57g | -0.31 | 0.15 |
| Lyn M Steffen, 2003 | IHD | 0-6g | 60-302g | -0.33 | 0.15 |
| Alysha S. Thompson, 2022 | IHD | 0-54g | 57-65g | -0.13 | 0.05 |
| Alysha S. Thompson, 2022 | IHD | 0-54g | 68-77g | -0.06 | 0.05 |
| Alysha S. Thompson, 2022 | IHD | 0-54g | 80-174g | -0.15 | 0.05 |
| Emily Sonestedt, 2015 | IHD | 0-0g | 0-9g | -0.16 | 0.08 |
| Emily Sonestedt, 2015 | IHD | 0-0g | 9-21g | -0.09 | 0.07 |
| Emily Sonestedt, 2015 | IHD | 0-0g | 21-36g | -0.16 | 0.08 |
| Emily Sonestedt, 2015 | IHD | 0-0g | 36-75g | -0.13 | 0.07 |

**Section 6. Sensetivity results**

Analysis results without trimming were presented in **Supplementary Figures 1-4**.

**Supplementary Figure 2**: Whole grain consumption and type 2 diabetes: Non-trimmed data.


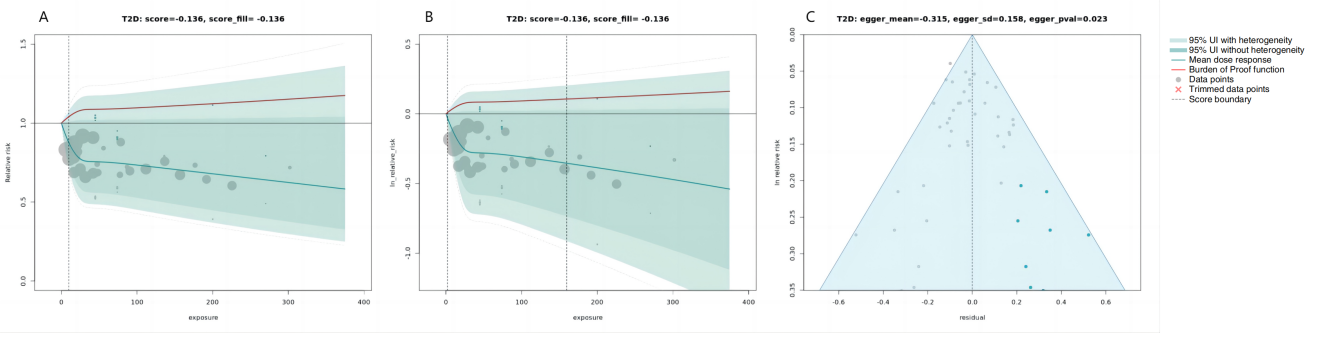


**A,** log-relative risk function

**B,** relative risk function

**C,** A modified funnel plot showing the residuals (relative to 0) on the x-axis and the estimated standard deviation

(SD) that includes reported SD and between-study heterogeneity on the y-axis

**Supplementary Figure 3**: Whole grain consumption and ischemic heart disease: Non-trimmed data.


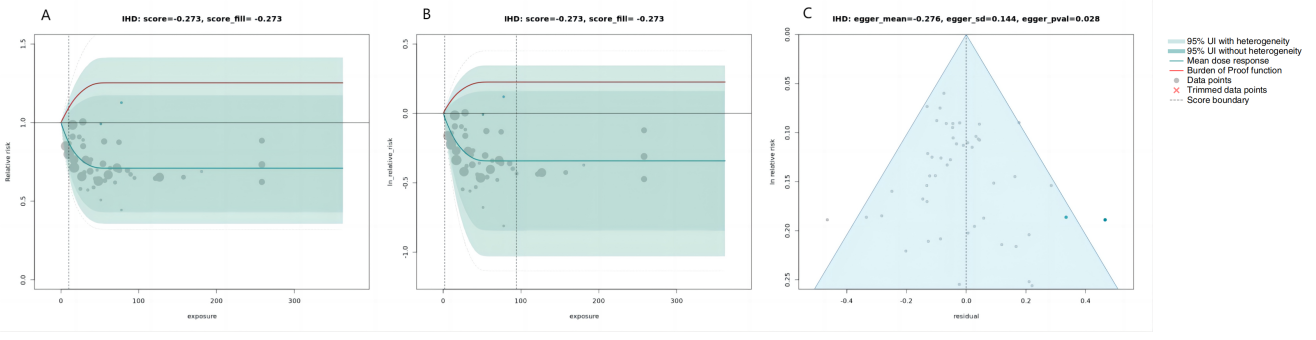


**A**, log-relative risk function

**B**, relative risk function

**C**, A modified funnel plot showing the residuals (relative to 0) on the x-axis and the estimated standard deviation

(SD) that includes reported SD and between-study heterogeneity on the y-axis

**Supplementary Figure 4**: Whole grain consumption and colorectal cancer: Non-trimmed data.


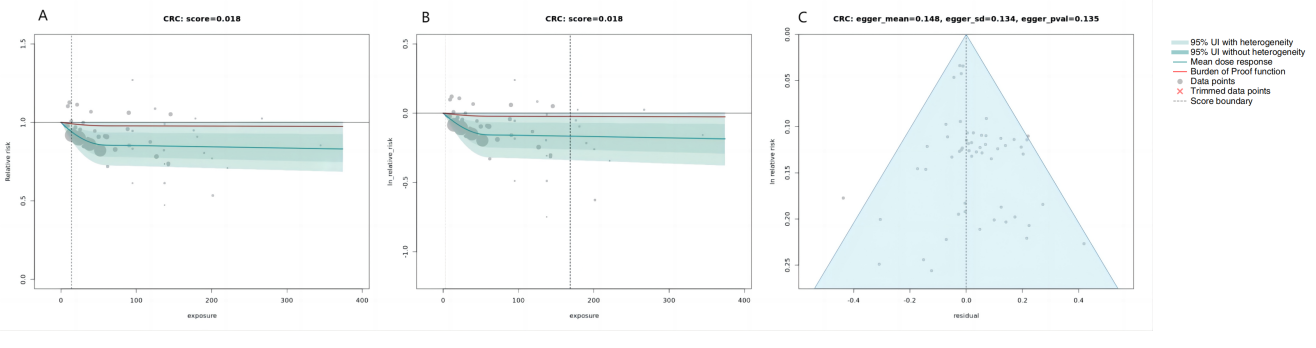


**A**, log-relative risk function

**B**, relative risk function

**C**, A modified funnel plot showing the residuals (relative to 0) on the x-axis and the estimated standard deviation

(SD) that includes reported SD and between-study heterogeneity on the y-axis

**Supplementary Figure 5**: Whole grain consumption and stroke: Non-trimmed data.


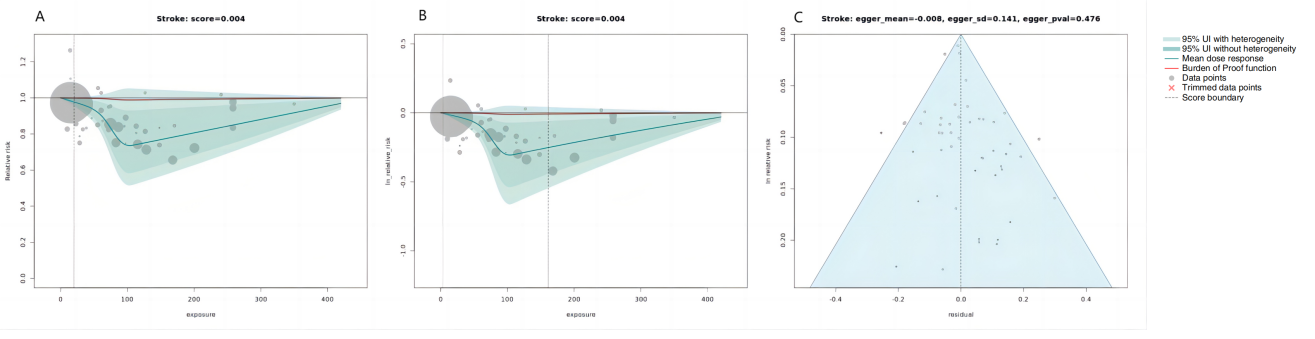


**A**, log-relative risk function

**B**, relative risk function

**C**, A modified funnel plot showing the residuals (relative to 0) on the x-axis and the estimated standard deviation

(SD) that includes reported SD and between-study heterogeneity on the y-axis
